# Epilepsy: Epidemiology, Molecular Pathogenesis, and Clinical Management

**DOI:** 10.1002/mco2.70837

**Published:** 2026-06-30

**Authors:** Jian Liu, Xinyan Wu, Ting Yin, Feining Huang, Manqi Kong, Yu Yang, Zinan Liu, Jiabin Yu, Tianpeng Zhang

**Affiliations:** ^1^ School of Pharmaceutical Sciences Guangzhou University of Chinese Medicine Guangzhou China; ^2^ Key Laboratory of Chinese Medicinal Resource from Lingnan (School of Pharmaceutical Sciences, Guangzhou University of Chinese Medicine) Ministry of Education Guangzhou China; ^3^ Institute of Molecular Rhythm and Metabolism Guangzhou University of Chinese Medicine Guangzhou China

**Keywords:** clinical management, epidemiology, epilepsy, mechanism

## Abstract

Epilepsy is a common neurological disorder with a substantial global burden. Despite major advances in diagnosis and therapy, nearly one‐third of patients remain resistant to antiseizure medications, highlighting persistent gaps in understanding epileptogenesis and disease progression. Here, we review epidemiological evidence, time‐dependent seizure patterns, and pathogenic mechanisms that contribute to epilepsy. We discuss how genetic variants, ion‐channel dysfunction, altered synaptic transmission, neuroinflammation, metabolic and mitochondrial stress, structural remodeling, network reorganization, and epigenetic regulation converge to destabilize neural circuits. These processes interact across disease stages and promote persistent hyperexcitability. We further summarize how mechanistic advances are reshaping clinical management, including precision diagnostics, pharmacotherapy, surgery, neuromodulation, dietary and lifestyle intervention, chronotherapy, biomarker‐guided stratification, and emerging disease‐modifying approaches such as immunotherapy, pathway‐targeted treatment, RNA‐based therapeutics, and gene‐directed strategies. Data‐driven tools for seizure detection and forecasting are also discussed as complementary approaches for individualized care. Overall, current evidence supports a shift from empirical seizure suppression toward mechanism‐guided and individualized care. Future progress will require closer integration of molecular discovery, validated biomarkers, and real‐world implementation to achieve earlier, more equitable, and potentially disease‐modifying treatment.

## Graphical abstract

Epilepsy is a heterogeneous and chronically evolving brain network disorder. This review integrates epidemiological burden, psychiatric comorbidities, and cyclic seizure patterns with multiscale pathogenic mechanisms, including ion‐channel dysfunction, synaptic transmission defects, neuroinflammation, metabolic and mitochondrial dysfunction, and epigenetic alterations. These interacting processes destabilize neural circuits and sustain chronic hyperexcitability. Advances in mechanistic understanding are reshaping clinical management, supporting mechanism‐guided and individualized strategies such as RNA‐ and gene‐based therapies, novel antiseizure drugs, immunotherapy, pathway‐targeted treatment, and biomarker‐guided intervention.

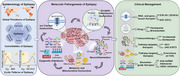

## 1 Introduction

Epilepsy is among the most common chronic neurological disorders, affecting more than 50 million individuals worldwide and contributing substantially to premature mortality, long‐term disability, and reduced quality of life [1, 2]. Defined by a persistent predisposition to generate epileptic seizures, it comprises a heterogeneous group of disorders with structural, genetic, metabolic, infectious, immune, and developmental causes [3]. The impact of epilepsy extends beyond recurrent seizures. Many patients experience psychiatric, cognitive, sleep‐related, and somatic comorbidities, as well as impaired educational attainment, employment disadvantage, social stigma, and reduced daily functioning [4]. These consequences also affect families, caregivers, and health systems, especially in low‐ and middle‐income countries (LMICs), where diagnostic capacity is limited and treatment gaps remain wide [5]. Given its chronic, recurrent, and multidimensional complexity, epilepsy remains a profound global neurological challenge that demands deeper mechanistic insights, accelerated diagnostics, and more efficacious and equitable interventions.

Over the past two decades, advances in molecular genetics, neuroimaging, and systems neuroscience have changed the way epilepsy is understood. Epilepsy was once viewed mainly as a disorder of single‐neuron hyperexcitability or focal structural lesions [6]. It is now increasingly recognized as a multiscale network disorder in which seizure generation, propagation, and persistence emerge from the interaction of abnormalities across molecular, cellular, circuit, and systems levels [7]. At the molecular level, rare pathogenic variants, somatic mutations, and polygenic risk can alter ion channels, neurotransmitter receptors, synaptic signaling, and transcriptional programs [8, 9]. These changes shift the balance between excitation and inhibition and increase circuit vulnerability. At the tissue and network levels, neuroinflammation, glial dysfunction, mitochondrial stress, metabolic disturbance, and maladaptive structural remodeling further destabilize neural circuits [10, 11]. These interacting processes help explain why seizures recur, propagate through distributed networks, evolve over time, and become drug resistant in many patients.

These pathogenic processes do not act in isolation. Rather, they form interdependent layers of epileptogenic dysfunction. Genetic susceptibility may alter ion‐channel function, synaptic transmission, and neuronal excitability [12]. These primary disturbances can then engage glial activation, inflammatory signaling, mitochondrial injury, and metabolic stress. In turn, immunometabolic dysregulation may further enhance neuronal hyperexcitability and promote maladaptive network remodeling [13]. Additionally, repeated seizures can strengthen these pathological changes through feed‐forward mechanisms, leading to more stable circuit reorganization and progressive disease consolidation [14]. Thus, epilepsy is best viewed as a systems disorder in which molecular, cellular, inflammatory, metabolic, and network‐level abnormalities converge to destabilize brain circuits over time.

This conceptual transition has important translational implications. More than 30 antiseizure medications (ASMs) are now available, yet approximately one‐third of patients still develop drug‐resistant epilepsy (DRE) [15, 16]. These patients remain at high risks of injury, cognitive decline, psychiatric comorbidity, and sudden unexpected death in epilepsy (SUDEP). Surgical resection, neuromodulation, dietary therapy, and immunotherapy have improved outcomes in selected populations [17]. However, these approaches are not appropriate for all patients and remain unevenly accessible. Importantly, most current treatments remain largely symptomatic. They suppress seizures but do not reliably prevent epileptogenesis, halt disease progression, or reverse established network pathology [18]. This limitation has prompted a shift toward mechanism‐guided intervention. Advances in genomic diagnostics, biomarker discovery, pathway‐targeted therapy, immunomodulation, antisense and RNA‐based therapeutics, and gene‐directed interventions are beginning to reshape epilepsy care [19]. The emerging goal is not only to reduce seizure frequency, but also to modify disease course and match treatment to the biological drivers of epilepsy in individual patients.

In this review, we discuss epilepsy from disease burden to biological mechanism and clinical management. We first summarize the epidemiology of epilepsy, including incidence, prevalence, etiological heterogeneity, comorbidity burden, and long‐term outcomes. This section defines the scale of the problem and the clinical needs that remain unmet. We then then review the main mechanisms implicated in epileptogenesis, focusing on genetic susceptibility, ion‐channel and synaptic dysfunction, neuroinflammation, metabolic and mitochondrial stress, epigenetic regulation, and structural and functional network remodeling. We further discuss how these biological insights are changing clinical management. Particular attention is given to precision diagnosis, mechanism‐based treatment, biomarker‐guided stratification, seizure forecasting, and emerging disease‐modifying strategies. Through this structure, we aim to provide a coherent framework for understanding epilepsy as a chronic network disorder and for translating mechanistic advances into more individualized and effective care.

## 2 Epidemiology of Epilepsy

### 2.1 Global Incidence and Prevalence

The International League Against Epilepsy (ILAE) defines epilepsy as at least two unprovoked seizures occurring more than 24 h apart, one unprovoked seizure with a long‐term recurrence risk of at least 60%, or diagnosis of a recognized epilepsy syndrome [20]. Epilepsy affects people of all ages, sexes, and regions. Its occurrence reflects the combined effects of genetic susceptibility, structural or metabolic brain abnormalities, developmental insults, infections, immune disturbances, and environmental factors. For epidemiological assessment, epilepsy is commonly described as lifetime epilepsy, active epilepsy, or incident epilepsy. These categories allow comparison of disease frequency, treatment needs, and health burden across populations and regions.

**FIGURE 1:**
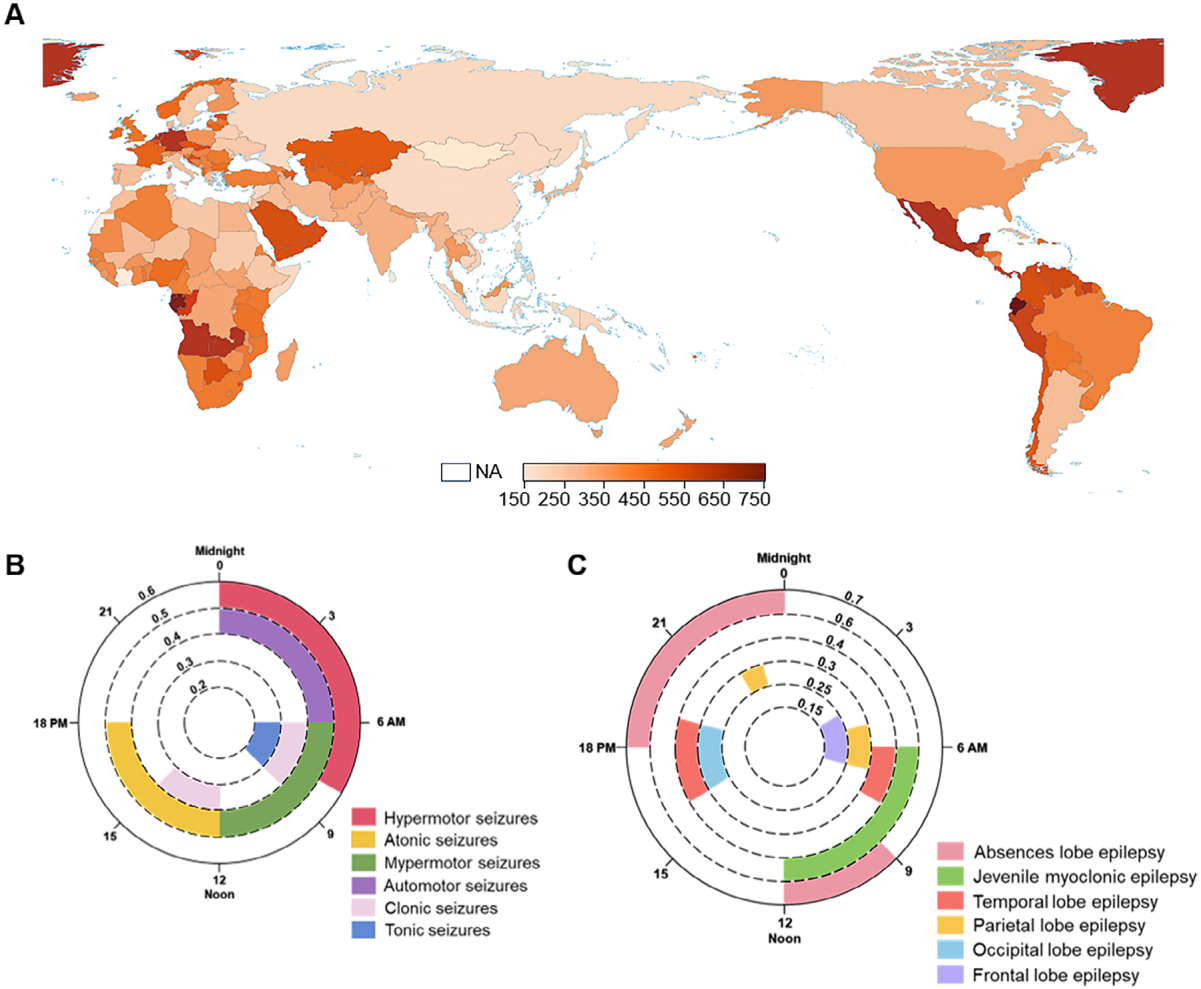
Prevalence and circadian patterns of epilepsy. (A) Global prevalence of epilepsy among adults across countries and regions in 2021 (Map approval number: GS (2016)1667). (B) Circadian phase distribution of seizure types. Hypermotor and automotor seizures primarily during the nocturnal period, while tonic and clonic seizures are more common during daytime and early evening. Atonic seizures peak near the day‐to‐evening transition. (C) Circadian phase distribution of major epilepsy syndromes. Juvenile myoclonic epilepsy and absence epilepsy peak from the early morning to daytime, whereas temporal lobe and frontal lobe epilepsies are enriched during nocturnal or sleep‐associated periods. Parietal and occipital lobe epilepsies show more restricted seizure timing within intermediate circadian phases.

According to the Global Burden of Disease 2021 estimates, there were 3.27 million new cases of idiopathic epilepsy worldwide in 2021, with a global age‐standardized incidence rate of 42.8 per 100,000 persons (Figure 1A) [2]. Incidence follows a U‐shaped age distribution, with peaks in early childhood and late adulthood, particularly among individuals aged 85 years and older [21]. Across most age groups, males have slightly higher incidence rates than females [22]. Epilepsy incidence also varies substantially across regions and socioeconomic settings. Age‐standardized incidence rates are highest in high‐sociodemographic index (SDI) regions in the GBD 2021 estimates (Figure 1A) [2]. However, meta‐analyses indicate that the absolute incidence of epilepsy remains greater in LMICs, with pooled estimates of approximately 61–70 cases per 100,000 person‐years, nearly twice the rates reported in high‐income regions [23]. These differences likely reflect the combined effects of perinatal injury, central nervous system infections, traumatic brain injury, environmental exposures, socioeconomic disadvantage, and unequal access to diagnosis and care. Racial and ethnic disparities further indicate that epilepsy risk is shaped not only by biological susceptibility, but also by clustered social and environmental determinants of health [24]. In parallel, genetic background contributes to the incidence pattern of specific epilepsy subtypes, particularly idiopathic generalized epilepsies (IGEs), which often show familial aggregation and earlier onset.

The pooled global point prevalence of active epilepsy is approximately 6.4 per 1000 population, whereas lifetime prevalence is estimated at about 7.6 per 1,000 [23]. Prevalence is consistently higher in LMIC, with rural LMIC regions reaching 10–15 per 1000, exceeding the 4–7 per 1000 typically observed in high‐income countries [25]. This disparity is also reflected in disability burden. Global Burden of Disease 2021 estimates indicate that low‐SDI regions have the highest age‐standardized disability‐adjusted life‐year rates, exceeding 300 per 100,000, compared with approximately 125 per 100,000 in high‐SDI regions [20]. The age pattern of prevalence differs from that of incidence. Prevalence generally increases through adolescence and early adulthood, declining during midlife, and remains relatively stable in later life [2]. Sex differences are modest, with slightly higher prevalence in males than in females [22]. Genetic epilepsies contribute substantially to lifetime prevalence, whereas structural and metabolic causes, including stroke, infection, and tumors, become more prominent in older adults and in LMICs [26]. In some regions, the burden is further increased by micronutrient deficiencies, neurotoxin exposure, and endemic parasitic infections [27]. Collectively, these demographic, geographic, occupational, nutritional, and genetic factors explain the marked heterogeneity of epilepsy burden worldwide [28].

### 2.2 Etiological Classification and Risk Factors

The 2017 ILAE framework and the 2024 World Health Organization update classify epilepsy causes into six, often overlapping, etiological categories, including structural, genetic, infectious, metabolic, immune, and unknown [29]. Structural causes include perinatal brain injury, stroke, traumatic brain injury, cortical malformations, tumors, and neurodegenerative disorders [3]. Genetic epilepsies encompass both monogenic variants and complex polygenic risk. More than 500 epilepsy‐associated genes have been identified, genetic causes account for approximately one‐third of early‐onset cases [30]. Infectious causes remain important worldwide and include neurocysticercosis, tuberculosis, and viral encephalitis [31]. Metabolic and immune epilepsies are increasingly recognized and include treatable inborn errors of metabolism and autoimmune encephalitis, such as anti‐NMDA receptor and LGI1 antibody‐mediated disorders [32]. Despite these advances, the cause of epilepsy remains unidentified in approximately half of patients worldwide, underscoring the need for broader diagnostic access and deeper mechanistic investigation.

Etiological categories are closely linked to risk factors that operate at different stages of life. In childhood and adolescence, population‐based systematic reviews identify perinatal complications, preterm birth, neonatal seizures, intracranial hemorrhage, and hypoxic–ischemic injury as major factors for epilepsy risk [33]. These factors are particularly important because early brain injury may alter neurodevelopmental trajectories and increase long‐term seizure susceptibility. In adults and older individuals, acquired structural injury, intracerebral hemorrhage, ischemic stroke, and central nervous system infections, are major causes of post‐traumatic and poststroke epilepsy [34]. In LMIC, recent studies further confirm the importance of head injury, perinatal insults, central nervous system infections, febrile seizures, poor sanitation, and limited access to perinatal care [35]. Many of these factors are potentially preventable, indicating that epilepsy prevention is partly dependent on public health infrastructure. Occupational and lifestyle exposures, including neurotoxic agents, heavy alcohol consumption, and recreational drug use, also contribute to epilepsy risk, particularly when combined with structural or genetic vulnerability [28]. Thus, epilepsy risk reflects the cumulative impact of developmental, structural, infectious, environmental, and behavioral factors across the life course.

### 2.3 Comorbidities and Long‐Term Outcomes

Epilepsy is frequently accompanied by psychiatric, neurological, and systemic disorders. Psychiatric conditions are particularly common. Approximately 30%–50% of people with epilepsy have at least one psychiatric disorder, most commonly depression, anxiety, sleep disorder, attention‐deficit/hyperactivity disorder, or psychosis [36]. Many show bidirectional relationships with epilepsy, whereby psychiatric symptoms increases seizure susceptibility, while uncontrolled seizures can worsen mood, cognition, sleep, and behavior [37]. Neurological comorbidities are also frequent. Migraine, stroke, traumatic brain injury, dementia, autism spectrum disorder, and cerebral palsy are among the most common associated conditions. Some reflect shared causes, such as early brain injury or genetic susceptibility, whereas others may be linked through common mechanisms, including neuroinflammation, altered network excitability, and impaired synaptic plasticity [38]. Multimorbidity is therefore a defining feature of epilepsy care. Large cohort studies show that 60%–70% of adults and about 80% of children with epilepsy have at least one chronic comorbidity, while more than half of children with developmental and epileptic encephalopathies (DEEs) have five or more [39]. Systemic diseases further add to this burden. Recent national datasets report higher rates of cardiovascular disease, metabolic disorders, gastrointestinal conditions, chronic respiratory disease, and cancer among adults with epilepsy [39]. These associations may reflect shared risk factors, reduced physical activity, social disadvantage, long‐term ASM exposure, and the biological effects of chronic epilepsy. Thus, epilepsy should be managed as a multidimensional disorder rather than as recurrent seizures alone.

Comorbidity is a major determinant of long‐term outcomes in epilepsy. Individuals with epilepsy and multimorbidity have higher rates of hospitalization, polypharmacy, cognitive decline, and reduced health‐related quality of life [40]. Recent cohort analyses demonstrate that psychiatric or somatic disorders increase the risk of DRE and accelerate functional decline [41]. Among these conditions, depression and anxiety are especially important. They are strong predictors of poor quality of life and are associated with increased suicide risk and poorer seizure control [38].

Premature mortality remains one of the most serious consequences of epilepsy. Long‐term studies consistently show that mortality is two‐ to threefold higher in people with epilepsy than in the general population in high‐income countries, with even greater excess mortality reported in LMICs [39]. In childhood‐onset epilepsy, mortality remains elevated over decades, and epilepsy‐related causes account for more than half of deaths in long‐term follow‐up [42]. Risk is highest in patients with structural etiologies, persistent seizures, nocturnal generalized tonic–clonic seizures, and failure to achieve sustained remission [43]. SUDEP is a major epilepsy‐related cause of death. International analyses estimate its incidence at approximately 1.2 per 1000 person‐year, with highest risk among individuals with persistent convulsive seizures and inadequate access to care [38]. Multimorbidity further increases mortality risk. In adults, many deaths are attributable to somatic diseases, particularly cardiovascular and pulmonary disease [43, 44]. These findings indicate that improving long‐term outcomes requires more than seizure suppression. Early seizure control, active identification and treatment of comorbidities, SUDEP risk reduction, and coordinated multidisciplinary care are central to reducing disability and premature death.

### 2.4 Cyclic Patterns of Epileptic Seizure

Seizures do not occur randomly across the 24‐h cycle. In many epilepsy syndromes, seizure occurrence shows a clear circadian pattern, although the preferred phase differs by seizure type and syndrome. Clinical studies have shown that hypermotor and automotor seizures tend to cluster during the night, and atonic seizures occur more often in the afternoon, whereas myoclonic, clonic, and tonic seizures occur more often in the morning (Figure 1B) [45, 46, 47]. Syndrome‐specific patterns are also evident. Frontal lobe epilepsy often shows an early morning predominance, occipital lobe epilepsy tends to peak in the late afternoon, and juvenile myoclonic epilepsy (JME) is characterized by seizures shortly after awakening (Figure 1C) [46]. Other syndromes, including temporal lobe epilepsy (TLE) and absence epilepsy (AE), may show bimodal circadian distributions [46, 48]. These temporal patterns are clinically relevant because they may inform seizure monitoring, risk prediction, and time‐of‐day‐adapted treatment. However, seizure timing is not fixed. It can vary within the same syndrome according to seizure subtype, age, ethnicity, sleep–wake state, treatment status, and disease stage.

Experimental models support a circadian regulation of seizure susceptibility. In mice and rats, pilocarpine‐ or kainic acid‐induced SE is followed by spontaneous recurrent seizures that show clear daily patterning, often with higher seizure frequency during the afternoon [49, 50]. Rodent models of SE and chronic TLE exhibit robust daily rhythms in seizure occurrence [51]. The phase of these rhythms differs from that observed in humans, which is consistent with the opposite activity phases of nocturnal rodents and diurnal humans. Nevertheless, tonic–clonic seizures in both humans and rodents show light‐associated temporal patterns, suggesting that photic entrainment may influence seizure probability across species [47]. Several mechanisms may underlie these temporal patterns. Circadian seizure patterns have been linked to clock regulated sleep–wake cycles, daily fluctuations in neuronal excitability, and rhythmic hormone secretion including melatonin, cortisol, and catecholamines [52, 53]. Sleep state is another key modifier. Some epilepsies are preferentially activated during nonrapid eye movement sleep, whereas others occur more often during wakefulness [54]. Sleep deprivation consistently lowers seizure threshold, indicating that seizure timing reflects the combined effects of circadian phase, sleep–wake architecture, and intrinsic network excitability [54, 55].

Seizure timing is not limited to the 24‐h cycle. Long‐term recordings show that many patients have multiday seizure cycles, often occurring at weekly or monthly intervals [56]. Circapseptan rhythms have also been observed in both humans and animal models, suggesting that seizure risk may be shaped by behavioral routines, stress exposure, treatment patterns, and endogenous biological cycles [56]. Seasonal variation provides another level of temporal organization. Seizure frequency may change with weather conditions, ambient temperature, humidity, sunlight exposure, and vitamin D status [57, 58]. These observations indicate that seizure occurrence is governed by multiple time scales. Together, these findings indicate that seizure timing is shaped by interacting circadian, multiday, seasonal, hormonal, and environmental factors, highlighting the importance of temporal biology for understanding epileptogenesis, improving seizure forecasting, and optimizing chronotherapy‐based clinical management.

### 2.5 Circadian Clock Dysregulation and Epilepsy Susceptibility

Accumulating genetic and experimental evidence support a role for circadian clock genes in epileptic vulnerability. Among them, *RORα* has the clearest developmental link. In humans, pathogenic variants in *RORα* are linked to intellectual developmental disorder, often accompanied by epilepsy, autism spectrum features, or cerebellar ataxia [59]. Whole‐exome sequencing has identified *RORα* variants in patients with developmental delay, and epilepsy, and rare deleterious variants occur significantly more often in individuals with DEE than in controls. A population study also associated the *RORα* rs12912233 polymorphism with epilepsy susceptibility in Malaysian Chinese cohorts, whereas *RORα* mutations appear uncommon in typical generalized or focal epilepsies [60]. Concordantly, *Rorα*‐deficient mice develop spontaneous seizures and cerebellar ataxia, accompanied by developmental defects in Purkinje cells [61]. These findings suggest that RORα may influence epilepsy risk by regulating cerebellar and broader circuit maturation.

Clock‐network perturbation studies further indicate that core circadian transcriptional regulators can directly shape seizure threshold and network excitability. Global *Clock* deletion exacerbates kainic acid induced TLE and is associated with changes in oxidative stress‐related pathways involving GPX4 and PPARγ [62]. Conditional loss of *Clock* in cortical pyramidal neurons reduces inhibitory postsynaptic currents, lowers seizure threshold, and induces spontaneous epileptiform discharges [62, 63]. These findings suggest that CLOCK may stabilize cortical networks, at least in part, by maintaining inhibitory synaptic tone and redox homeostasis. The CLOCK partner BMAL1 also modulates seizure susceptibility. Global *Bmal1* deficiency lowers electroshock thresholds, whereas dentate gyrus‐specific deletion of *Bmal1* shortens seizure latency, potentially via reduced protocadherin‐19 expression [64]. Downstream clock‐controlled genes further modify epileptic phenotypes. Loss of *Rev‐erbα* attenuates seizure severity and frequency in acute and chronic TLE models, whereas deletion of the PAR bZIP factors (*Hlf/Dbp/Tef*) results in lethal spontaneous epilepsy [65, 66]. Together, these studies show that circadian clock genes do more than time seizure occurrence. They also influence neuronal inhibition, oxidative stress, synaptic organization, and circuit stability, thereby modifying epileptic susceptibility and disease severity.

Modern lifestyles that disrupt sleep and circadian timing may influence epilepsy risk and seizure control. In a UK Biobank analysis of approximately 270,000 participants followed for more than a decade, usual or permanent night shift work was associated with a higher incidence of epilepsy [67]. Poor sleep quality also showed a graded relationship with epilepsy risk [67]. Adverse sleep traits, including short or long sleep duration, insomnia, and daytime sleepiness, tended to be associated with increased risk [68]. These findings are consistent with long‐standing clinical observations. Circadian misalignment and sleep loss lower seizure threshold and worsen seizure control, particularly in individuals with insomnia or irregular schedules. Longitudinal patient‐level datasets further suggest that sleep disruption may precede seizures in a dose‐dependent manner. Even modest sleep curtailment relative to an individual baseline has been linked to a short‐term increase in next‐day seizure probability [68]. Wearable‐based and home monitoring studies provide additional support, showing that sleep timing, sleep efficiency, and seizure occurrence are often coupled in daily life. However, the strength and direction of these associations vary across epilepsy syndromes and individuals.

Evidence linking jet lag and long‐haul travel to seizure exacerbation is less consistent and is often confounded by sleep debt, medication nonadherence, stress, and alcohol exposure. Nonetheless, clinical observations suggest that rapid time‐zone shifts may precipitate breakthrough seizures in susceptible patients, especially in those with frequent baseline seizures [69]. Light exposure represents a distinct trigger in a smaller group of patients. In visually provoked or photosensitive epilepsy, intermittent photic stimulation and real‐world sources such as strobe lighting or some video games can induce seizures under permissive conditions [70]. Sleep and circadian disruption may also downstream risks. They can increase nocturnal seizure propensity and aggravate injury, cognitive impairment, and mood symptoms. Because nocturnal generalized tonic–clonic seizures are strongly associated with SUDEP risk, stabilizing sleep may be relevant to risk reduction, although direct causal evidence remains limited [68]. These findings support the clinical value of sleep continuity and circadian‐aligned scheduling as low‐cost adjuncts to antiseizure therapy. However, prospective studies are still needed to define causal effects, syndrome‐specific vulnerability, and the patients most likely to benefit from circadian or sleep‐focused intervention.

Together, epilepsy is a globally prevalent and heterogeneous neurological disorder. Its burden varies by age, sex, region, and socioeconomic context, shaped by interacting genetic, structural, infectious, metabolic, immune, and environmental determinants. Epidemiological and experimental evidence demonstrates that seizure occurrence follows robust circadian, multiday, and seasonal rhythms, and that modern lifestyle factors, particularly sleep deprivation, circadian misalignment, shift work, and abnormal light exposure, may increase seizure susceptibility and worsen seizure control in vulnerable patients.

## 3 Molecular Pathogenesis of Epilepsy

Epileptogenesis is increasingly understood as a progressive process rather than the result of a single pathogenic pathway [7]. Genetic architecture establishes a susceptible background by altering ion channels, synaptic proteins, intracellular signaling, and neurodevelopmental programs [71]. These genetic liabilities are translated into cellular level as abnormal intrinsic excitability and impaired synaptic transmission, which shift E/I balance toward recurrent firing [72]. Once seizures emerge, neuronal hyperactivity further recruits glial, inflammatory, and vascular responses and imposes substantial metabolic demand [73]. This links excessive firing to immunometabolic dysfunction. With repeated seizures, these processes drive synaptic rewiring, local circuit remodeling, and large‐scale network reorganization, thereby converting episodic hyperexcitability into more persistent epileptic connectivity [73]. Epigenetic mechanisms further stabilize this state by transforming transient activity and inflammation‐dependent signals into stable transcriptional alterations [74]. Thus, epilepsy emerges from nested feed‐forward loops in which genetic, synaptic, immune, metabolic, and structural abnormalities interact dynamically across disease stages to drive seizure onset, epileptogenesis, disease progression, and drug resistance.

### 3.1 Genetic Architecture

Genetic architecture provides an upstream basis for epileptogenesis. Across epilepsy syndromes, pathogenic variants confer baseline susceptibility to circuit instability. Their effects are usually expressed through downstream processes, including ion‐channel dysfunction, abnormal synaptic transmission, disturbed developmental signaling, and altered cellular metabolism [75]. Thus, epilepsy genetics is important not only because it explains heritability, but also because it identifies molecular entry points through which diverse causes converge on shared pathogenic pathways.

**FIGURE 2:**
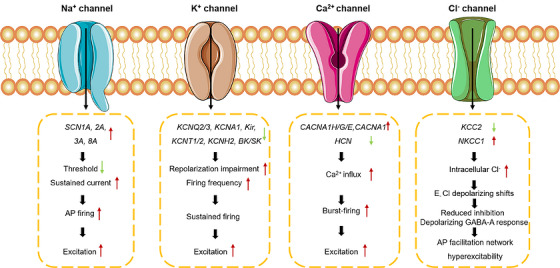
Ion‐channel dysfunction in epilepsies. Upregulation of voltage‐gated sodium‐channel genes (*SCN1A*, *SCN2A*, *SCN3A*, and *SCN8A*) lowers the activation threshold and increases sustained Na^+^ current, thereby boosting action‐potential firing and overall neuronal excitation. Downregulation of multiple potassium‐channel genes (*KCNQ2/3*, *KCNA1*, *KCNT1/2*, *Kir*, *KCNH2*, *BK*, and *SK*) impairs repolarization, increases firing frequency, and drives sustained firing and neuronal excitation. Increased expression of calcium‐channel genes (*CACNA1H, CACNA1G*, and *CACNA1*) together with reduced HCN activity increases Ca^2^^+^ influx and burst firing. Decreased *KCC2* and increased *NKCC1* raise intracellular Cl^−^, depolarize E_Cl, and convert GABA_A_‐mediated signaling from inhibitory to depolarizing.

Epilepsy spans a broad genetic continuum, from highly penetrant monogenic disorders to complex polygenic forms shaped by numerous variants of modest effect. Large‐scale exome and genome sequencing studies have established that rare, ultra‐rare, and common variants all contribute to disease risk, with their relative influence differing across DEEs, genetic generalized epilepsies (GGEs), and focal epilepsies (Figure 2A) [76]. In a cohort of 20,979 individuals undergoing exome sequencing, six genes, including *SCN1A* and *DEPDC5*, reached exome‐wide significance [77]. Gene‐set analyses highlighted voltage‐gated sodium and potassium channels and GABA_A_ receptor complexes as major contributors to genetic liability [77]. Complementary genome‐wide association meta‐analyses involving nearly 30,000 cases identified 26 common‐variant risk loci, predominantly associated with GGE, and demonstrated that common variants account for approximately 40%–90% of heritable liability across GGE subtypes [78]. Together, these findings support a graded model of epilepsy genetics. Severe early‐onset epilepsies are often driven by rare, highly penetrant variants, whereas common epilepsies usually reflect the combined effects of rare variants, common variants, and nongenetic modifiers.

A substantial proportion of early‐onset and severe epilepsies are caused by pathogenic variants in single genes regulating ion channels, synaptic transmission, intracellular signaling pathways, and cellular metabolism. Among them, ion‐channel genes are the best characterized. Variants in *SCN1A*, *SCN2A*, and *KCNQ2* cause overlapping but distinct phenotypic spectra, ranging from neonatal DEEs and Dravet syndrome to self‐limited familial epilepsies [79]. Genotype–phenotype studies have refined this view. In *SCN1A*, variant type and location are associated with disease severity and pharmacological responsiveness. Similar principles apply to *SCN2A* and *KCNQ2*. Early‐onset gain‐of‐function variants often cause neonatal DEEs and may respond to sodium‐channel blockers. By contrast, later‐onset loss‐of‐function variants are more often associated with milder epilepsies or broader neurodevelopmental disorders [80, 81]. Monogenic epilepsies are not limited to ion‐channel disorders. Pathogenic variants also affect synaptic vesicle proteins (STXBP1 and SNAP25), neurotransmitter receptors, mTOR‐pathway regulators (DEPDC5 and TSC1/2), and metabolic enzymes. These genes account for 30%–50% of previously unexplained DEEs and an increasing fraction of familial epilepsies [82].

Copy‐number variants (CNVs) and other structural variants represent another important source of genetic risk in epilepsy. Recurrent microdeletions and microduplications at loci, such as 15q11.2, 15q13.3, 16p11.2, 1q21.1, and 22q11.2, confer several‐fold increases epilepsy risk, typically accompanied by broader neurodevelopmental phenotypes [83]. Chromosomal microarray and CNV sequencing studies report pathogenic CNV yields of 2%–15% in unselected epilepsy cohorts, rising to more than 20% in individuals with developmental delay or dysmorphic features [84]. Family‐based analyses further demonstrate that both recurrent and private structural variants contribute to familial epilepsies [85]. Many of these variants overlap with CNVs implicated in autism and intellectual disability, underscoring shared developmental mechanisms. Thus, CNV analysis is particularly valuable when epilepsy occurs with neurodevelopmental impairment or syndromic features.

Beyond rare high‐impact variants, common variants and polygenic risk scores (PRS) increasingly capture the multifactorial inheritance of common epilepsies. Higher PRS increases lifetime epilepsy risk, particularly in IGEs and earlier‐onset disease [86]. A 2025 meta‐analysis found that individuals in the top 0.5% of the PRS distribution show approximately 4.6‐fold increased odds of GGE and 1.7‐fold higher odds of focal epilepsy [87]. Polygenic burden may also modify epilepsy arising from acquired brain injury. Among 17,549 stroke survivors from the UK Biobank, those in the highest decile of the epilepsy PRS had nearly fivefold higher odds of generalized poststroke epilepsy and approximately threefold higher odds of focal poststroke epilepsy than those in the lowest decile [88]. PRS may also help explain phenotypic variability within families. Family‐based studies further demonstrated that PRS can modulate the penetrance and severity of rare pathogenic variants, providing a mechanistic explanation for intrafamilial phenotypic variability despite shared monogenic mutations [89].

These findings support an integrated genetic architecture in which rare monogenic variants, structural variation, and polygenic background interact to shape individual susceptibility, age at onset, and clinical course. Genetic findings are therefore no longer limited to explaining heritability. They are increasingly used to refine syndromic classification, establish etiological diagnosis, guide prognosis, inform recurrence counseling, and support treatment selection [90]. Genotype‐defined epilepsies also provide a practical framework for precision medicine by identifying patients who may benefit from pathway‐targeted interventions, mechanism‐informed ASM choices, or emerging gene‐directed therapies. These include antisense oligonucleotides (ASOs), transcriptional modulation, and viral gene replacement strategies [90]. More broadly, incorporating rare pathogenic variants, copy‐number variation, somatic mosaicism, and polygenic susceptibility into clinical evaluation may enable earlier diagnosis and improved stratification of DEEs [91]. In this way, epilepsy care is beginning to move beyond phenotype‐based management toward biologically grounded intervention.

### 3.2 Ion‐Channel Dysfunction

Ion‐channel dysfunction is a core mechanism of epileptic hyperexcitability. Voltage‐gated sodium (Na_V_), potassium (K_V_, K_Ca_, and K_Na_), calcium (Ca_V_), and chloride‐handling channels alter action‐potential threshold, firing pattern, repolarization kinetics, and inhibitory restraint. Disturbance of these channels can convert genetic or acquired susceptibility into neuronal hyperexcitability at the level of single neurons and local microcircuits (Figure 2) [92].

Sodium‐channel disorders illustrate this principle most clearly. In monogenic epilepsies, recurrent seizures commonly result from reduced Na_v_ function in inhibitory interneurons or excessive function in excitatory neurons (Figure 2) [93]. Loss‐of‐function variants in *SCN1A*, including truncating and missense mutations, reduce Na_v_1.1 currents in parvalbumin‐ and somatostatin‐positive interneurons, impairing inhibitory firing and leading to disinhibition of pyramidal neurons [94]. Mouse models and induced pluripotent stem cell models consistently support interneuron hypoexcitability as a major driver of Dravet syndrome [95, 96]. This mechanism provides a rationale for therapies that enhance Na_v_1.1 function or strengthen GABAergic inhibition. In contrast, gain‐of‐function variants in *SCN2A* and *SCN8A* increase persistent sodium current and promote burst firing in cortical pyramidal neurons [97]. These DEEs often respond favorably to sodium‐channel blockers such as phenytoin, underscoring the therapeutic value of functionally informed genetic classification [93].

Potassium‐ and calcium‐channel dysfunction provides another major route to epileptic hyperexcitability. Variants in *KCNA1*, *KCNQ2/3*, *KCNT1*, and *KCNB1* are associated with neonatal epilepsies, early‐onset epileptic encephalopathies, and progressive myoclonus epilepsy (Figure 2) [95]. Their effects depend strongly on channel subtype and cellular context. Impaired potassium‐mediated repolarization in interneurons reduces inhibitory output, whereas excessive potassium conductance in pyramidal neurons can precipitate firing failure and network instability [98]. Calcium channelopathies exert similarly complex effects. T‐type Ca_v_3.2 and Ca_v_3.3 channels, which support rhythmic burst firing, are strongly implicated in generalized epilepsies and absence seizures (Figure 2) [99]. Advances in structural biology and pharmacology targeting Ca_v_2.3 and Ca_v_3.2 are now guiding the development of next‐generation small‐molecule and antisense therapies [100]. Presynaptic Ca_v_2.1 channels, encoded by *CACNA1A*, play a pivotal role in coupling action potentials to synaptic vesicle fusion, and both gain‐ and loss‐of‐function variants disrupt neurotransmitter release probability and synaptic strength [101]. Notably, redirecting alternative Ca_v_ isoforms to the active zone can restore vesicle release, suggesting that presynaptic channel engineering may become a future therapeutic strategy [102]. Chloride handling is also critical for inhibitory control. Downregulation of KCC2 or upregulation of NKCC1 increase intracellular Cl^−^, depolarize E_‐_Cl, and weakens GABA_A_‐mediated inhibition [103]. Under these conditions, GABAergic signaling may shift from hyperpolarizing inhibition toward depolarizing excitation, thereby promoting network instability.

Collectively, sodium, potassium, and calcium channelopathies disrupt neuronal firing, repolarization, alter presynaptic calcium entry, neurotransmitter release, and inhibitory restraint. This explains why similar seizure phenotypes may require different therapeutic strategies. Treatment choice depends on whether a variant produces gain or loss of function, alter persistent or transient currents, and preferentially affect excitatory neurons, inhibitory interneurons, or presynaptic release machinery [92]. Ion‐channel biology therefore provides one of the most actionable frameworks in epilepsy. It supports function‐informed ASM selection, development of subtype‐selective channel modulators, and emerging gene‐directed approaches designed to restore physiological channel function rather than broadly suppress seizures [104].

### 3.3 Synaptic Transmission Dysfunction

**FIGURE 3:**
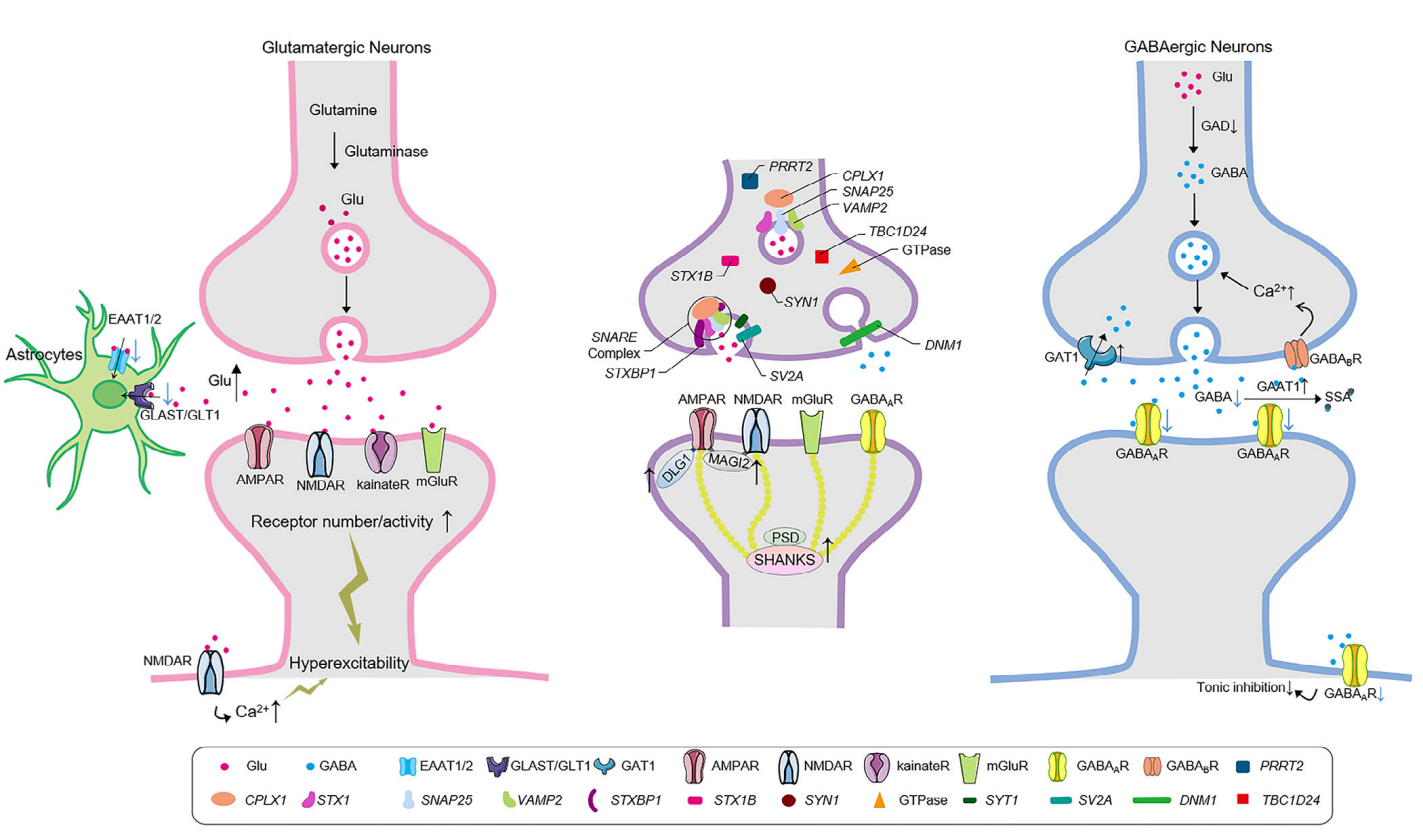
Synaptic transmission dysfunction in epilepsy. Disruption of glutamatergic and GABAergic signaling is a central driver of epileptic hyperexcitability. Impaired astrocytic glutamate clearance through EAAT1/2 dysfunction, combined with dysregulation of AMPA, NMDA, metabotropic glutamate, and kainate receptors, increases extracellular glutamate and amplifies excitatory drive. Persistent activation of extrasynaptic NMDA receptors further promotes Ca^2^^+^ influx, membrane depolarization, and seizure generation. Inhibitory transmission is concurrently weakened by reduced GABA synthesis, impaired vesicular loading and reuptake, and downregulation of GABA_A_ receptor subunits. Presynaptic abnormalities that impair vesicle priming, fusion, endocytosis, and replenishment additionally disturb neurotransmitter release and short‐term synaptic plasticity, promoting pathological network synchrony.

Synaptic transmission dysfunction is a key step through which neuronal hyperexcitability becomes network‐level epileptic activity. Ion‐channel defects may alter the firing properties of individual neurons, but seizures emerge only when abnormal activity is transmitted, amplified, and synchronized across synaptic circuits [105]. Therefore, disruption of excitatory and inhibitory neurotransmission is central to epileptogenesis. In physiological states, the synthesis, release, and clearance of glutamate and γ‐aminobutyric acid (GABA) are tightly controlled, whereas in epilepsy these processes are markedly disrupted. At excitatory synapses, impaired glutamate clearance, particularly due to astrocytic *EAAT1/2* dysfunction, leads to extracellular glutamate accumulation. Together with altered glutamate‐receptor composition, this prolongs depolarization, increases Ca^2+^ influx, and promotes excitotoxic signaling (Figure 3) [106]. At inhibitory synapses, reduced GABA synthesis, impaired vesicular loading, abnormal reuptake, receptor remodeling, and disturbed chloride homeostasis weaken both phasic and tonic inhibition [107, 108]. Presynaptic abnormalities further destabilize synaptic transmission by impairing vesicle docking, priming, fusion, and recycling, especially in fast‐spiking inhibitory interneurons. Together, glutamatergic overdrive, weakened GABAergic inhibition, and presynaptic release defects shift E/I balance toward excitation, lower seizure threshold, and promote epileptogenesis.

Glutamatergic overactivation is further amplified by dysregulation of ionotropic glutamate receptors, including AMPA, NMDA, and kainate receptors, at the levels of expression, trafficking, subunit composition, and synaptic localization [109]. Studies of epileptic tissue have shown increased GluA1/2 turnover, upregulation of the NMDA receptor subunit *GluN2B*, and altered localization of the kainate receptor subunit *GluK2* (Figure 3) [110, 111]. These alterations prolong depolarization, increase Ca^2^^+^ influx, and enhance excitotoxic signaling. Experimental models confirm that excessive receptor activation disrupts intracellular homeostasis, and accelerates epileptogenesis [112]. Consistently, neuroimaging and surgical pathology studies in humans reveals region‐specific increases in AMPA and NMDA receptor activity in hippocampal sclerosis and focal cortical dysplasia [113]. Impaired inhibitory control is another major driver of epileptic network instability. It may result from interneuron loss, reduced GABA synthesis, reduced GABA_A_ receptor subunit expression, remodeling of GABA_B_ receptor subunit/isoform, and impaired chloride homeostasis [114]. Genetic evidence supports this mechanism. Pathogenic variants in multiple GABA_A_ receptor subunit genes (e.g., *GABRA1/2/3/5*, *GABRB1/2/3*, *GABRD*, and *GABRG2*) are linked to epilepsy syndromes including Dravet syndrome, Lennox–Gastaut syndrome, and febrile seizure phenotypes [115]. GABA_A_ and GABA_B_ receptors constrain excitability through complementary mechanisms. GABA_A_ receptors mediate fast phasic inhibition, whereas GABA_B_ signaling provides slower and longer lasting restraint by suppressing presynaptic neurotransmitter release and enhancing postsynaptic inhibition [116]. Experimental studies support a seizure‐suppressive role for presynaptic GABA_B_ signaling. For example, low‐frequency electrical stimulation reduces 4‐aminopyridine‐evoked epileptiform activity via presynaptic GABA_B_‐dependent mechanisms [117]. Clinically, electrophysiological recordings from TLE tissue demonstrate attenuated inhibitory postsynaptic potentials, underscoring the importance of GABAergic dysfunction in seizure initiation and propagation [118].

Presynaptic dysfunction is an important contributor to synaptic imbalance in epilepsy. Pathogenic variants affecting synaptic vesicle loading, docking, priming, fusion, and recycling are increasingly identified in monogenic epilepsies [119]. Loss‐of‐function mutations in *SYN1*, *STXBP1*, and *DNM1* disrupt vesicle pool replenishment, reduce the readily releasable pool, impair synchronous neurotransmitter release, and exaggerate short‐term synaptic depression (Figure 3) [120]. These defects are particularly detrimental to fast‐spiking inhibitory interneurons, particularly parvalbumin‐positive cells, because these neurons require sustained and precisely timed transmitter release to maintain network inhibition (Figure 3) [121]. STXBP1 encephalopathy provides a clear clinical example. It is characterized by early‐onset seizures, developmental impairment, and burst‐suppression EEG patterns [122]. *STXBP1* encodes Munc18‐1, a key regulator of vesicle docking and fusion. Haploinsufficiency impairs transmitter release at both inhibitory and excitatory synapses, but preferential failure of inhibition appears to be a dominant driver of epileptogenesis [123]. Thus, presynaptic release defects can produce epilepsy not simply by reducing synaptic transmission globally, but by selectively weakening the inhibitory control required to stabilize local circuits.

In addition to glutamate and GABA, modulatory transmitter systems also shape seizure threshold. Acetylcholine, dopamine, norepinephrine, serotonin, histamine, orexin, and neuropeptide Y regulate excitability by tuning E/I balance, network synchrony, autonomic tone, and arousal state. Cholinergic signaling is strongly proconvulsant in limbic circuits, as illustrated by pilocarpine‐induced status epilepticus and associated disruption of muscarinic receptor pathways [124]. In contrast, dopaminergic and noradrenergic signaling are often protective, although their effects depend on receptor subtype and circuit context. D2‐like dopamine signaling can be anticonvulsant, whereas reduced norepinephrine tone increases seizure vulnerability [125, 126]. Serotonergic signaling also regulate neuronal excitability and seizure‐linked autonomic function, with potential relevance to SUDEP [127]. Among neuropeptides, neuropeptide Y acts as an activity‐dependent brake on seizure propagation, whereas orexin links arousal, sleep architecture, and limbic excitability, making it a candidate target in sleep‐sensitive epilepsies [128, 129]. Histaminergic signaling is generally anticonvulsant in several paradigms, with H3 receptors emerging as a pharmacologic lever that can influence both seizures and cognition [130]. These transmitter systems indicate that epileptic networks are shaped not only by fast synaptic excitation and inhibition, but also by slower neuromodulatory control of brain state, arousal, and circuit gain.

Together, these findings identify synaptic dysfunction as a major mechanism of epileptogenesis. Abnormal transmitter release, impaired glutamate clearance, excessive glutamate‐receptor activation, weakened GABAergic inhibition, and defective vesicle cycling all disturb E/I balance. These changes promote seizure initiation, facilitate seizure propagation, and contribute to persistent network instability. This mechanism has direct therapeutic relevance. Several current ASMs already act on synaptic targets, including GABAergic transmission, glutamate receptors, and SV2A‐dependent vesicle release [131]. Differences in synaptic pathology may also help explain why patients with similar seizure phenotypes respond differently to treatment. For example, seizures driven mainly by impaired inhibition may require a different therapeutic strategy from those dominated by glutamatergic overactivation or presynaptic release defects. A more precise understanding of synaptic pathology may further enable circuit‐selective interventions that restore inhibitory control, limit excitotoxic recruitment, or normalize maladaptive synaptic plasticity [132]. Synaptic mechanisms therefore remain central to biomarker discovery, refinement of antiseizure pharmacology, and the development of future disease‐modifying therapies.

### 3.4 Neuroinflammation and Immune Mechanisms

**FIGURE 4:**
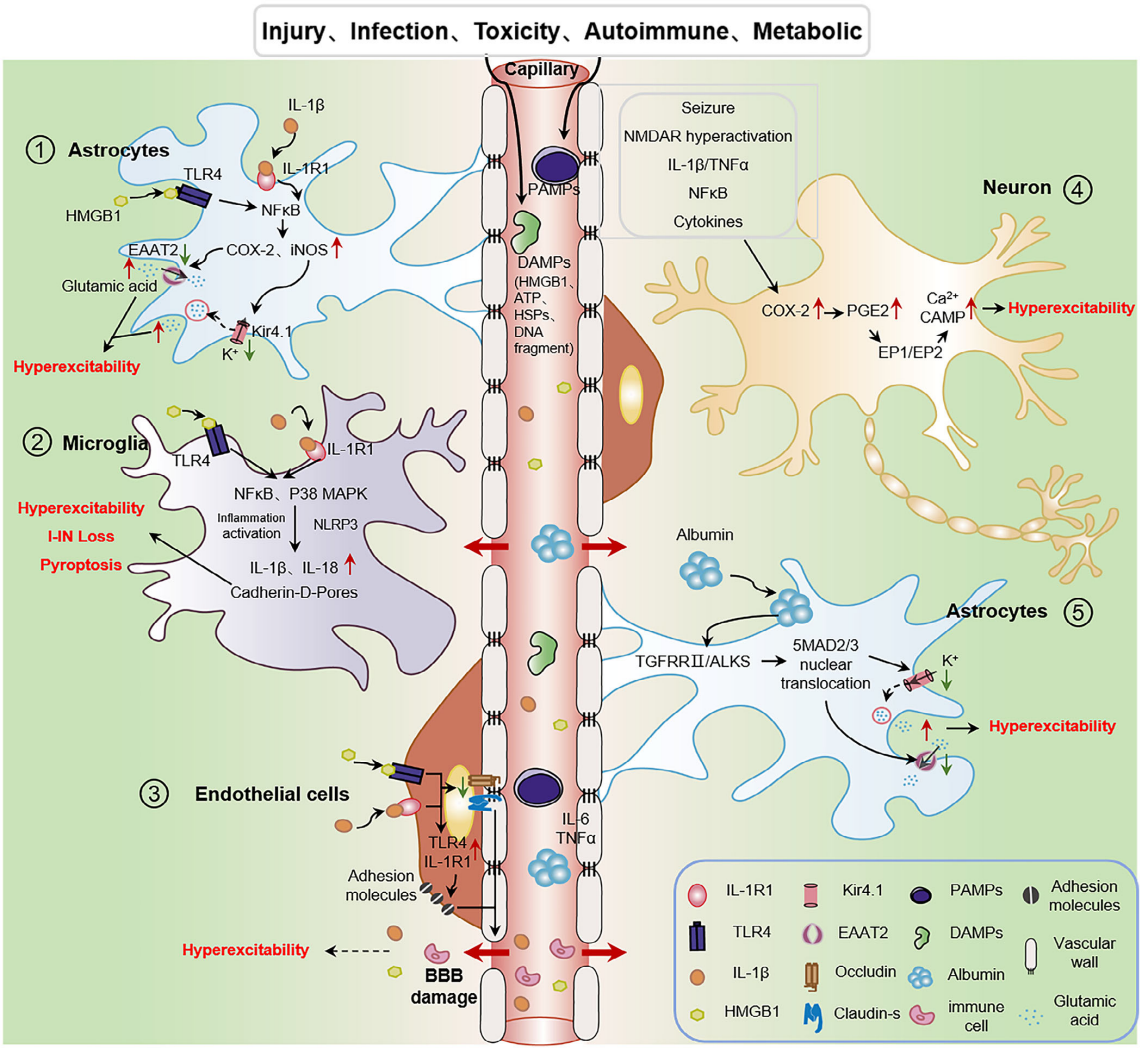
Inflammatory mechanisms driving epileptic hyperexcitability. In astrocytes, HMGB1‐TLR4 and IL‐1β‐IL‐1R1 signaling activate NF‐κB, promoting proinflammatory mediators, COX‐2, and iNOS while suppressing EAAT2 and Kir4.1, thereby disrupting glutamate clearance and K^+^ buffering. In microglia, the same pathways engage NF‐κB and p38 MAPK, and activate the NLRP3 inflammasome, promoting IL‐1β and IL‐18 release, gasdermin‐D pore formation, pyroptosis, interneuron loss, and network hyperexcitability. In endothelial cells, HMGB1 and IL‐1β increase TNF‐α, IL‐6, and adhesion molecule expression through TLR4 and IL‐1R1, thereby disrupting the BBB and facilitating immune‐cell infiltration. Seizures, NMDAR overactivation, and inflammatory mediators also induce COX‐2‐dependent PGE_2_ synthesis. EP1/EP2 signaling increases intracellular Ca^2^^+^ and cAMP to further enhance excitability. BBB leakage additionally permits albumin entry, which activates astrocytic TGF‐β‐ALK5‐SMAD2/3/4 signaling, further suppresses EAAT2 and Kir4.1, and reinforces recurrent hyperexcitability.

Once recurrent hyperactivity is established, neuroinflammation becomes both a consequence and a driver of epileptogenesis. Diverse brain insults, including excessive neuronal firing, tissue injury, blood–brain barrier (BBB) disruption, and glial activation can rapidly initiate innate immune responses, including HMGB1‐TLR4, IL‐1β‐IL‐1R1, NF‐κB, STAT3, inflammasome, and TGF‐β signaling (Figure 4) [133]. These inflammatory cascades induce the production of IL‐1β, TNF‐α, IL‐6, prostaglandins, complement components, and other mediators [134]. Such factors can lower seizure threshold by altering ion‐channel function, enhancing glutamatergic transmission, weakening inhibitory control, promoting vascular inflammation, and increasing BBB permeability. In this way, immune activation does not merely follow seizures. It feeds back onto neuronal and vascular compartments and reinforces the ion‐channel and synaptic defects that sustain network instability [135]. Experimental studies support this pathogenic role. Blockade of IL‐1R/TLR signaling, NLRP3 inflammasome activation, or COX‐2/EP2 pathways, attenuates epileptogenesis and seizure severity in multiple models [136].

BBB dysfunction provides a critical link between inflammation and epileptogenesis. Seminal studies showed that leakage of serum albumin extravasation into brain parenchyma activates astrocytic TGF‐β receptor‐SMAD signaling [137]. This pathway downregulates Kir4.1 and AQP4, impairs potassium and glutamate buffering, promotes excitatory synaptogenesis and facilitates recurrent seizures (Figure 4). Clinical and experimental studies have extended this mechanism to acquired epilepsies. In poststroke and post‐traumatic epilepsy, early BBB leakage is accompanied by albumin or fibrinogen deposition, and increased expression of TGF‐β, matrix metalloproteinase‐9, and adhesion molecules [138]. Importantly, seizures can in turn aggravate BBB disruption. Excessive neuronal activity promotes Ca^2+^‐dependent cytoskeletal remodeling, ion‐channel dysfunction, and mislocalization of AQP4 and Kir4.1 [139]. This creates a feed‐forward loop in which barrier breakdown, glial activation, leukocyte entry, cytokine release, impaired ion homeostasis, and neuronal hyperexcitability reinforce one another (Figure 4).

Adaptive immune responses add layer to neuroimmune regulation in epilepsy. They are most evident in autoimmune and infection‐related epilepsies, but may also contribute to epilepsies with other etiologies. In autoimmune encephalitis, B‐cell and plasma‐cell responses generate antibodies against neuronal surface and synaptic antigens, including NMDAR, GABABR, AMPAR, GluR3, and LGI1 [140]. These antibodies disrupt receptor trafficking, synaptic signaling, and neuronal excitability, thereby creating highly epileptogenic limbic circuits. A proportion of patients subsequently develop chronic autoimmune‐associated epilepsy [141]. T‐cell‐mediated cytotoxicity and complement activation have also been detected in resected epileptic tissue across different etiologies, indicating sustained adaptive immune engagement involvement beyond classic autoimmune encephalitis [142]. Antibodies such as GluR3B can alter CD4^+^/CD8^+^ T‐cell ratios and elevate circulating cytokines, further supporting a role for adaptive immunity in modulating seizure burden [143]. These immune responses may persist long after the initial insult. By maintaining microglial activation, cytokine production, and maladaptive synaptic remodeling, they may help stabilize epileptic networks over time [32].

Chronic neuroinflammation is increasingly viewed as an active contributor to epileptogenesis, seizure progression, and long‐term comorbidities. Longitudinal TSPO‐PET imaging and cerebrospinal fluid (CSF) biomarker studies demonstrate persistent microgliosis and astrogliosis in epileptogenic regions. These changes are accompanied by sustained increases in HMGB1, IL‐1β, TNF‐α, CCL2, IL‐33, and related inflammatory mediators [144, 145]. Their levels correlate with seizure frequency, drug resistance, and cognitive decline. Elevated serum or CSF levels of HMGB1, TLR4, TNF‐α, or CCL2 are associated with more severe and refractory disease [146]. Inflammation also intersects with metabolic and synaptic mechanisms. Sustained cytokine signaling impairs mitochondrial function, disrupts redox homeostasis, and promotes maladaptive synaptic plasticity [13, 147]. These changes reinforce hyperexcitable networks and may contribute to neurodegeneration. Together, innate immune activation, BBB dysfunction, adaptive immune responses, and chronic glial signaling form interacting loops that can drive epileptogenesis, disease progression, and pharmacoresistance.

Clinically, these findings support biomarker‐guided identification of patients who may benefit from immunotherapy or anti‐inflammatory pathway modulation. In autoimmune epilepsies, early recognition of antibody‐ or T‐cell‐mediated pathology can directly alter management by favoring early immunotherapy, improving seizure control and potentially preventing chronic network reorganization [142]. More broadly, evidence implicating IL‐1, TNF, HMGB1‐TLR4, or TGF‐β signaling supports the development of pathway‐targeted anti‐inflammatory strategies in selected patients [133]. Inflammatory mediators detected in serum, CSF, and molecular imaging studies may serve as biomarkers for disease activity, treatment response, and risk stratification [146]. Thus, neuroinflammation should not be viewed only as a consequence of seizures. It is a mechanistically important and increasingly actionable component of epilepsy pathogenesis.

### 3.5 Metabolic and Mitochondrial Dysfunction

Metabolic and mitochondrial dysfunction represents a major amplifier of epileptogenesis. Recurrent neuronal firing imposes a high energetic demand. Na^+^/K^+^‐ATPase and Ca^2+^ pumps must continuously restore ionic gradients after repeated depolarization, whereas inflammation and oxidative stress impair the metabolic pathways required to sustain this demand [148]. The result is an energetic mismatch. ATP depletion reduces ion‐pump activity, aggravates mitochondrial injury, disrupts redox balance, and alters substrate utilization. These changes further destabilize membrane potential, neurotransmitter release, and glial homeostasis.

Experimental studies suggest a biphasic metabolic response. During acute seizure or SE‐like activity, glycolysis and oxidative phosphorylation increase transiently, reflecting a compensatory attempt to meet sudden energy demand [149]. Chronic epilepsy reduces ATP production capacity and respiratory‐chain protein abundance by 25%–40%. Fiber photometry experiments further demonstrate that hippocampal seizures induce an abrupt decline in neuronal ATP levels accompanied by increased astrocytic pyruvate, indicating a transient breakdown of neuron‐glia metabolic coupling [150]. Computational models of *GLUT1* deficiency corroborate support this mechanism. Poststimulus ATP depletion and Na^+^/K^+^‐ATPase failure are sufficient to generate seizure‐like after discharges, whereas restoration of glucose supply or modest enhancement of neuronal ATP normalizes excitability [151]. Insufficient ATP promotes ion pump failure, extracellular K^+^ accumulation, membrane depolarization, and enhanced glutamate release, collectively promoting hypersynchrony and ictal activity [152]. Clinical PET and metabolic imaging studies show a parallel pattern, with ictal hypermetabolism followed by chronic interictal hypometabolism [153]. These findings support an energy‐failure model in which recurrent ATP insufficiency can both trigger seizures and contribute to long‐term epileptogenesis.

Mitochondrial dysfunction adds a structural and molecular basis to this energetic vulnerability. Defects in respiratory‐chain complexes I, III, and IV, arising from nuclear or mitochondrial DNA mutations, are strongly associated with severe epilepsy [154]. Impaired electron transport increases mitochondrial reactive oxygen species (ROS) production, which damages ion channels, neurotransmitter receptors, membrane lipids, and mitochondrial DNA, further reducing respiratory efficiency and synaptic reliability [155]. Mitochondria also regulate intracellular homeostasis. Under physiological conditions, they buffer large Ca^2+^ loads during neuronal firing. In epilepsy, however, fragmented or overloaded mitochondria release Ca^2+^ back into the cytosol, prolonging depolarization, and promoting excitotoxicity [156]. Chronic seizures further disrupt mitochondrial dynamics by shifting the fission–fusion balance toward fragmentation, impairing mitophagy, and allowing dysfunctional organelles to accumulate in hippocampal and cortical neurons [157]. These alterations correlate with neuronal loss, hippocampal sclerosis, cognitive decline, and DRE.

Metabolic signaling pathways translate energetic stress into durable changes in neuronal excitability. The best‐characterized example is hyperactivation of mTOR signaling in mTORopathies, such as tuberous sclerosis complex and FCD type II [158]. Somatic mutations in *MTOR*, *TSC1/2*, *RHEB*, *DEPDC5*, and *PTEN* enhance protein synthesis, promote abnormal neuronal growth, and drive excessive synaptogenesis, generating enlarged and intrinsically hyperexcitable networks [159]. Preclinical studies demonstrate that mTOR inhibition reduces seizures and partially normalizes cortical architecture, establishing mTOR as a central metabolic regulator of epileptogenesis [160]. Other metabolic pathways also influence seizure susceptibility. AMP‐activated protein kinase (AMPK) senses cellular energy status through changes in ATP/AMP ratios. Acute AMPK activation can suppress excitability during metabolic stress, whereas chronic epilepsy is associated with maladaptive AMPK signaling and persistent energy imbalance [149]. Adenosine provides another endogenous brake on excitability through A1 receptors. In chronic epilepsy, this anticonvulsant tone is weakened by increased adenosine kinase expression and reduced adenosine availability [161]. Metabolic dysfunction and neuroinflammation further amplify one another, forming a loop in which mitochondrial stress promotes inflammatory activation, and inflammation further impairs energy metabolism [162].

Together, these finding place metabolic and mitochondrial dysfunction at the center of epileptogenesis and highlight them as therapeutic targets. mTORopathies provide a clear example. Mechanistic insight has enabled pathway‐targeted treatment with mTOR inhibitors in selected patients, establishing a model for precision therapy in epilepsy [159]. Similarly, impaired brain energy utilization in *GLUT1* deficiency support the use of ketogenic therapy and related metabolic interventions as mechanism‐based treatments rather than empiric adjuncts [163]. More broadly, oxidative stress, adenosine dysregulation, mitochondrial injury, and inflammation‐metabolism crosstalk help define biologically distinct subgroups of refractory epilepsy. Metabolic biomarkers and therapies aimed at restoring cellular energetics, redox balance, mitochondrial quality control, or glial metabolism may therefore open new avenues for disease modification.

### 3.6 Structural and Network Reorganization

Persistent hyperexcitability, neuroinflammation, and metabolic stress drive progressive structural and network remodeling in the epileptic brain [13, 73]. This remodeling is particularly evident in mesial TLE, where neuronal loss, gliosis, and synaptic rewiring progressively distort hippocampal architecture [164]. Mossy fiber sprouting, interneuron loss, dendritic remodeling, and aberrant synaptogenesis create local circuits with enhanced recurrent excitation and weakened inhibition. Over time, these local changes are incorporated into broader structural and functional connectomes, reshaping seizure spread and contributing to impairment, disease progression, and drug resistance [165].

Hippocampal sclerosis provides the best‐characterized example of this process. Classic pathological features include selective loss of CA1 and CA3 pyramidal neurons, degeneration of dentate hilar mossy cells, granule cell layer abnormalities, and marked gliosis, resulting in a disorganized and atrophic hippocampus [166]. High‐resolution MRI and quantitative microstructural studies further demonstrate that TLE with hippocampal sclerosis is associated with widespread cortical abnormalities extending beyond the hippocampus, and that early hippocampal neurodegeneration or dentate gyrus reorganization predict later chronic epilepsy and cognitive decline [167]. At the synaptic level, mossy fibers from dentate granule cells aberrantly sprout into the inner molecular layer and granule cell layer, forming recurrent excitatory synapses onto neighboring granule cells, ectopic granule cells, and basal dendrites [168]. Rodent models and analyses of human epileptic tissue consistently show that mossy cells loss and extensive mossy fiber sprouting promotes spontaneous recurrent seizures, with the degree of sprouting correlating with long‐term seizure burden [169].

Structural remodeling reshapes the functional E/I balance of epileptic circuits. In TLE and related experimental models, specific GABAergic interneuron populations are selectively vulnerable. These include parvalbumin‐ and somatostatin‐expressing interneurons, which normally innervate pyramidal neuron somata, axon initial segments, and distal dendrites [170]. Loss or dysfunction of these cells weakens both feedforward and feedback inhibition in the dentate gyrus and CA1–CA3 circuits [166]. Although interneuron dysfunction may be sufficient to initiate epileptogenesis, surviving interneurons often partially compensation. As a result, the epileptic network is not characterized by complete inhibitory failure, but by a chronically unstable and near‐critical E/I state [171]. Concurrently, sprouted mossy fibers and aberrant CA3–CA1 collateral connections form recurrent excitatory loops. These circuits can be activated by minimal input and rapidly recruit local networks. Optogenetic and electrophysiological studies further show that reorganized dentate circuits are intrinsically hyperexcitable [172]. Dynamic recordings further reveal that seizures are preceded by a gradual shift of the local E/I balance toward excitation, accompanied by disruption of interneuron‐mediated gamma and beta oscillations [173]. Thus, microstructural remodeling and interneuronopathy converge to produce hypersynchronous microcircuits that are highly susceptible to runaway activity.

Local circuit abnormalities are embedded within larger epileptic networks. Structural covariance and diffusion MRI connectome analyses indicate that both TLE and IGE deviate from normal small‐world network architecture, but in different ways [174]. In TLE, orbitofrontal–temporal–limbic subnetworks exhibit increased clustering and longer path length. This pattern suggests a more segregated and lattice‐like topology, with strengthened short‐range connectivity and reduced global integration. Graph‐theoretical studies across multiple cohorts further show topological regularization of gray‐matter networks in TLE [175]. Vulnerable hubs are often concentrated in mesial temporal structures and default‐mode regions. These changes may constrain information flow, facilitate recurrent seizure propagation, and contribute to memory and cognitive dysfunction. IGE shows a different network pattern. Across frontotemporal and parietal regions, clustering and path length are often reduced, consistent with a more randomized organization. Recent multisite and pediatric studies further identify syndrome‐specific generalized epilepsy networks characterized by altered modularity, hub reorganization, and reduced small‐world efficiency [176]. These network changes also align with spatial gradients of epilepsy risk gene expression, linking genetic susceptibility with systems‐level vulnerability. Longitudinal and postsurgical connectomic studies provide translational support for this network view. Seizures tend to propagate along pre‐existing white‐matter pathways. During ictal states, structure–function coupling increases, indicating that anatomical connectivity constrains seizure spread. Targeted resection or neuromodulation of key network hubs can partially restore global efficiency and improve seizure control [174]. Thus, epilepsy is not only a disorder of local lesions or microcircuits, but also a disorder of large‐scale network organization.

These findings support a multiscale model of epileptic network remodeling. Microstructural changes generate hyperexcitable local circuits. Interneuron loss or dysfunction destabilizes E/I balance. Large‐scale network reorganization then embeds these abnormalities into distributed epileptic connectomes. This perspective helps explain why epilepsy often extends beyond a visible lesion, why seizure propagation varies across patients, and why cognitive and psychiatric comorbidities differ even among individuals with similar seizure types. This network view has direct clinical relevance. Lesion location alone is often insufficient to predict surgical outcome, because seizure generation and propagation depend on the organization of the broader epileptic network. Structural and functional network analyses are therefore increasingly used for presurgical evaluation, risk stratification, and identification of epileptic hubs. These hubs may serve as targets for resection, ablation, or neuromodulation. Recognizing epilepsy as a disorder of pathological network reorganization also emphasizes the importance of timing. Recurrent seizures can consolidate maladaptive circuitry and increase the likelihood of drug resistance. Earlier intervention may therefore prevent local circuit abnormalities from becoming fixed within larger epileptic networks. In this sense, connectomic and circuit‐level insights are beginning to reshape epilepsy treatment from lesion‐centered control toward network‐informed intervention.

### 3.7 Epigenetic Alterations

Epigenetic dysregulation provides a mechanism by which transient pathogenic signals are consolidated into durable epileptogenic programs. Seizure activity, inflammatory signaling, and altered metabolic flux can rapidly reshape miRNA expression, DNA methylation, and histone modifications [74]. These changes regulate genes involved in ion transport, synaptic transmission, inflammation, mitochondrial function, and cellular survival. In this context, epigenetic changes are not merely downstream correlates of seizures, but it can stabilize the pathological transcriptional state produced by genetic susceptibility, excitability defects, neuroinflammation, and metabolic stress.

**FIGURE 5:**
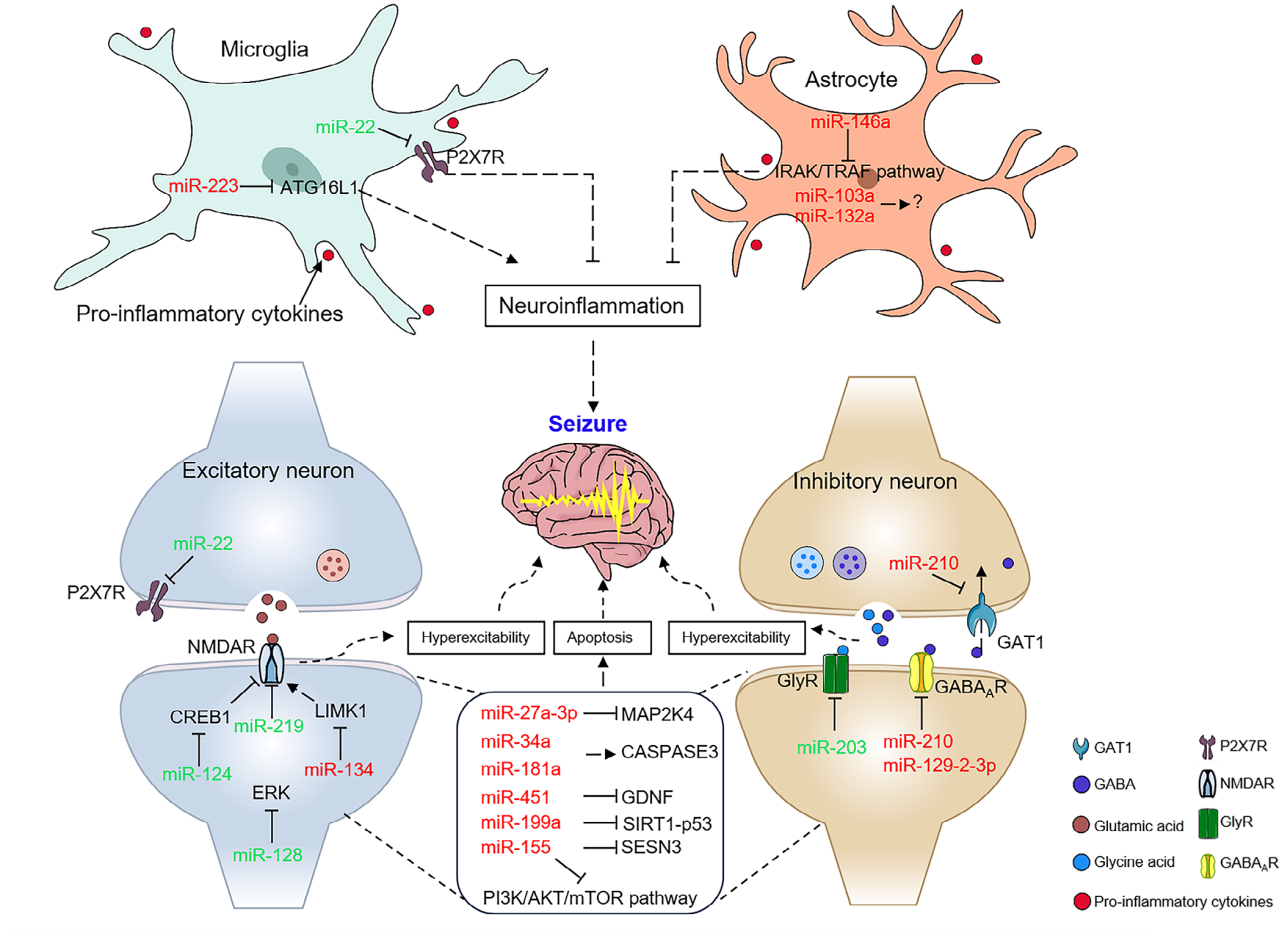
Role of mircoRNAs in epilepsy. Epileptogenic insults alter miRNA expression, with upregulated miRNAs (red) repressing target mRNAs and downregulated miRNAs (green) relieving repression of previously inhibited transcripts. These shifts disrupt signaling pathways that control dendritic structure, synaptic plasticity, neuronal excitability, and cell survival. In neurons, dysregulated miRNAs affect key mediators such as ERK, LIMK1, NRSF, and SIRT1, perturbing cytoskeletal organization, transcriptional regulation, and activity‐dependent remodeling. In astrocytes and microglia, altered miRNA networks enhance inflammatory and cytokine‐responsive signaling, thereby exacerbating neuroinflammation and network hyperexcitability.

miRNA dysregulation is one of the best‐studied epigenetic mechanisms in epilepsy. In mesial TLE with hippocampal sclerosis (MTLE‐HS), hippocampal tissue shows broad changes in microRNA expression. Many of these miRNAs are predicted to affect potassium channels, GABAergic signaling, axon‐guidance and synaptic organization, linking epigenetic remodeling to hyperexcitability and circuit reorganization (Figure 5) [177]. Experimental and clinical studies further demonstrate that seizures rapidly alter specific miRNAs, including miR‐132, miR‐134, miR‐124, and miR‐146a, which regulate dendritic spine morphology, synaptic plasticity, apoptosis, and inflammatory signaling (Figure 5) [178]. Among them, miR‐134 has received particular attention. Antisense inhibition of miR‐134 consistently suppresses spontaneous recurrent seizures and neuronal loss in multiple TLE models, highlighting anti‐miR‐134 oligonucleotides as promising disease‐modifying therapies (Figure 5) [179]. Circulating miRNAs are also being explored as minimally invasive biomarkers. Plasma miR‐134 and multi‐miRNA panels in pediatric epilepsy may help with diagnosis, prognosis, and therapeutic monitoring, although further validation in large prospective cohorts is still needed [180].

DNA methylation provides a more stable epigenetic substrate that helps maintain the epileptic state over time. Genome‐wide methylation analyses in TLE demonstrate widespread hypermethylation across coding and noncoding genomic regions, including miRNA and long noncoding RNA loci [181]. These changes are enriched in pathways related to ion channel, MAPK, and neurotrophin signaling and other processes involved in neuronal excitability and plasticity. In MTLE‐HS, extensive differentially methylated regions have been identified in both hippocampal and neocortical tissues, particularly within genes involved in inflammation. Importantly, methylation at many CpG sites appears to increase with disease duration, supporting the idea that epigenetic changes evolve during epileptogenesis rather than merely reflecting prior injury [182]. Similar methylation signatures in peripheral blood and cell‐free DNA also suggest potential as liquid biomarkers, although their specificity and clinical utility require further validation [183]. Mechanistic studies link DNA methylation to metabolic regulation. Adenosine deficiency and adenosine kinase upregulation drive excessive DNA methyltransferase activity and promote global hypermethylation [184]. Conversely, restoring adenosine signaling in animal models normalizes DNA methylation and can prevent epilepsy development [184].

Histone modifications provide an additional layer of epigenetic control by linking neuronal activity to enduring transcriptional programs. Seizures can disturb the balance between histone acetyltransferases and histone deacetylases (HDACs). In MTLE tissue and experimental models, Class I and II HDACs, including HDAC1, HDAC2, and HDAC5, are upregulated [185]. This shift is associated with reduced acetylation at promoters of genes involved in neuronal survival, inhibitory transmission, and anti‐inflammatory responses. Pharmacological HDAC inhibition in pilocarpine‐ and kainate‐induced epilepsy models attenuates seizure severity and delays or prevents the development of spontaneous seizures [74]. These findings suggest that HDAC signaling is not only a marker of epileptic tissue, but a modifiable contributor to epileptogenesis. Seizure‐induced alterations in histone methylation further refine gene regulation. One example is loss of the repressive H3K9me2 mark at the *Kcnj10* promoter, which alters potassium‐channel expression and may affect extracellular potassium buffering [186]. Emerging evidence also implicates histone lactylation as a mechanism linking metabolic flux, HDAC activity, chromatin accessibility, and seizure susceptibility [187].

Collectively, miRNA dysregulation, DNA methylation, and histone modifications form an interconnected epigenetic network in epilepsy. These mechanisms can convert transient seizure activity, inflammation, and metabolic stress into more durable transcriptional states. They may therefore contribute to both the development and maintenance of epileptic networks. Translationally, this is important because epigenetic alterations are potentially measurable and, in some cases, reversible. Epigenetic changes may serve as biomarkers for diagnosis, prognosis, disease monitoring, and treatment response [177]. They may also provide therapeutic entry points. Anti‐miRNA approaches, adenosine‐based modulation of DNA methylation, and chromatin‐targeted strategies such as HDAC inhibition are being explored as ways to modify epileptogenic programs rather than only suppress seizures [188]. Thus, epigenetic dysregulation links mechanism, biomarker discovery, and precision treatment in epilepsy.

### 3.8 Relevant Preclinical and Clinical Evidence in Epilepsy Pathogenesis

Preclinical and clinical studies demonstrate that epileptogenesis emerges from molecular disturbances that destabilize neuronal excitability and network homeostasis (Table 1). Animal models are particularly useful for testing causality. Human genetic, pathological, imaging, and biomarker studies provide translational support. Ion‐channel disorders provide the clearest example. In rodent models, loss‐of‐function mutations in *SCN1A* or *KCNQ2* produce hallmark features of DEEs, including interneuron hypoexcitability, impaired inhibitory control, and spontaneous seizures (Table 1) [80, 81]. These findings parallel clinical observations in Dravet syndrome and neonatal epilepsies, where pathogenic variants in the same genes lead to early‐onset, often pharmacoresistant epilepsy [80]. Similarly, gain‐of‐function variants in *SCN2A* and *SCN8A* enhance persistent sodium currents and promote neuronal bursting in experimental systems, consistent with the severe excitability phenotypes in affected patients (Table 1) [95]. Such cross‐species concordance supports ion‐channel dysfunction as a causal mechanism of epilepsy rather than a secondary epiphenomenon of seizures.

**TABLE 1:** Relevant preclinical and clinical evidence in epilepsy pathogenesis.

Mechanistic domain	Target	Key findings in animal models	Key findings in clinical studies	Proepileptogenic mechanism	References
Genetic architecture	*SCN1A* (Nav1.1)	Loss of *SCN1A* induces Dravet‐like phenotypes increased SUDEP susceptibility	Pathogenic *SCN1A* variants cause Dravet syndrome and DEEs	Interneuron hypoexcitability leads to cortical disinhibition and network hyperexcitability	[80, 81]
Genetic architecture	*SCN2A* (Nav1.2)	GOF variants enhance persistent sodium current and bursting, whereas LOF variants disrupt AP initiation and circuit maturation	*SCN2A* variants span neonatal‐onset DEEs to later‐onset epilepsy	Excitatory neuron firing instability and maladaptive plasticity → seizure initiation and progression	[81]
Ion‐channel dysfunction	*SCN8A* (Nav1.6)	GOF mutations increase neuronal firing and epileptiform activity; sodium‐channel blockers reduce seizures	*SCN8A*‐related DEEs is often severe and pharmacoresistant	Enhanced Na^+^ persistent current → high‐frequency firing → network hyperexcitability	[81, 97]
Ion‐channel dysfunction	*KCNQ2* (Kv7.2)	Loss of M‐current causes neonatal seizures; Kv7 openers reduce excitability	*KCNQ2* variants cause neonatal epilepsies/DEEs	Reduced subthreshold K^+^ current → impaired spike‐frequency adaptation → repetitive firing	[95]
Ion‐channel dysfunction	*KCNA1* (Kv1.1)	Kv1.1 deficiency leading to seizures	*KCNA1* variants can include epilepsy alongside EA1 phenotype	Repolarization deficit → increased presynaptic Na^+^ entry → glutamate release	[95]
Ion‐channel dysfunction	*KCNQ2/3* (Kv7.2/3)	Kv7 dysfunction increases repetitive firing; channel openers reduce seizures	Strongly associated with neonatal‐onset epilepsies	Reduced M‐current → membrane depolarization and hyperexcitability	[95]
Ion‐channel dysfunction	*KCNB1* (Kv2.1)	Loss‐of‐function causes intrinsic excitability changes and seizures in models.	*KCNB1* variants cause DEEs	Delayed rectifier K^+^ deficit → impaired repolarization → burst propensity	[95]
Ion‐channel dysfunction	*KCND2* (Kv4.2)	Altered seizure thresholds	Variants linked to neurodevelopmental disorders with epilepsy	Dendritic repolarization deficit → enhanced backpropagation and activity‐dependent plasticity	[95]
Synaptic transmission dysfunction	*STXBP1* (Munc18‐1)	Presynaptic release failure causes network hypersynchrony and early‐onset seizures	*STXBP1*‐related DEEs	Impaired vesicle docking/fusion → disruption inhibitory transmission	[120, 123]
Synaptic transmission dysfunction	EAAT2	Reduced EAAT2 level in epileptogenic tissue; EAAT2 upregulation‐induced suppression of seizure	Reduced EAAT2 function in epileptogenic hippocampus	Glutamate spillover → NMDA/AMPA overactivation → excitotoxicity and epileptogenesis	[106]
Synaptic transmission dysfunction	GAD65/67	Reduced GAD expression after SE	Reduced GAD in epileptic hippocampus reported; interneuron loss	Lower GABA synthesis → diminished inhibitory tone	[108]
Synaptic transmission dysfunction	GluK2	Modulation dentate and CA3 excitability and mossy fiber transmission	Hippocampal delivery of GluK2‐containing receptors can suppress seizures in TLE patients	Reinforces recurrent excitation in hippocampal circuits	[110]
Synaptic transmission dysfunction	Synaptic vesicle cycle proteins	Variants affecting docking, priming, Ca^2+^ sensing, recycling converges on impaired synaptic release	Vesicle cycle genes are enriched in epilepsy genetics and DEEs syndromes	Presynaptic failure → network disinhibition and synchrony	[120]
Neuroinflammation and immune	IL‐1β / IL‐1R1	Elevated IL‐1β signaling in multiple epileptic models; pharmacologic IL‐1R blockade reduces epileptogenesis	Elevated IL‐1β signaling in TLE/HS tissue and biofluids	Cytokine‐mediated modulation of glutamatergic receptors enhances excitability	[136, 146]
Neuroinflammation and immune	TNF‐α / TNFR1	Elevated TNF‐α level in multiple epileptic models; TNFR1 blockade reduces seizure burden	TNF signaling upregulated in epileptogenic tissue	TNF‐mediated receptor trafficking and glial activation → E/I imbalance	[145, 146]
Neuroinflammation and immune	HMGB1 / TLR4	Increased HMGB1‐TLR4 signaling; pathway blockade suppresses seizures	Elevated HMGB1 and TLR4 in epileptic tissue and circulation	TLR4→NF‐κB → proinflammatory cascade and synaptic modulation → epileptogenesis	[145, 146]
Neuroinflammation and immune	TGF‐β / ALK5 (BBB–albumin pathway)	Albumin extravasation activates astrocytic TGF‐β signaling	BBB dysfunction and albumin leakage in DRE	BBB breakdown → astrocyte TGF‐β → impaired K^+^/glutamate buffering → hyperexcitability	[137, 139]
Structural and network reorganization	Mossy fiber sprouting (DG)	Post‐SE sprouting forms recurrent excitatory loops	Hallmark in MTLE‐HS tissue	Recurrent excitation and reduced inhibition → self‐sustaining microcircuits	[167, 168]
Metabolic and mitochondrial dysfunction	*DEPDC5* (GATOR1)	Loss increases mTOR signaling and induces focal seizures and cortical malformations	Familial focal epilepsy; somatic second‐hit in FCD	mTORC1 disinhibition → aberrant growth and epileptogenic circuit formation	[160]
Metabolic and mitochondrial dysfunction	TSC1/2	Hyperactive mTOR signaling drives seizures; mTOR inhibitors reduce seizure	TSC‐associated epilepsy; everolimus reduces seizures	mTORC1 hyperactivation → abnormal neuronal/glial growth and excitatory connectivity	[160]
Metabolic and mitochondrial dysfunction	MTOR	Somatic MTOR GOF induces FCD‐like lesions and seizures in models.	Somatic MTOR variants in FCD type II and hemimegalencephaly; linked to DRE	mTOR‐driven dysplasia → hyperexcitable networks	[160]
Metabolic and mitochondrial dysfunction	*GLUT1* (SLC2A1)	*GLUT1* deficiency increases seizure susceptibility; ketosis rescues phenotype	*GLUT1* deficiency syndrome	Energy substrate failure → Na^+^/K^+^ pump stress and excitability increase	[151, 192]
Metabolic and mitochondrial dysfunction	Na^+^/K^+^‐ATPase	ATP depletion impairs pump → depolarization and extracellular K^+^ rise	Pump dysfunction in some genetic epilepsies; hypometabolism in epileptic foci	Ion homeostasis failure → depolarization, glutamate release and synchrony	[152, 153]
Epigenetic alterations	miR‐134	Antagomir or ASOs inhibition reduces seizures and neuronal loss in TLE models	Upregulated in epileptic tissue; circulating biomarker in MTLE‐HS	Regulation of spine density and synaptic plasticity → maladaptive rewiring	[178]
Epigenetic alterations	miR‐132	Upregulated miR‐132 expression following seizures	Elevated in epileptic tissue and biofluids	Activity‐dependent transcriptional program → strengthened excitatory connectivity	[178]
Epigenetic alterations	miR‐146a	Elevated in epileptic tissue	Upregulated in epileptic tissue	Modulation TLR/IL‐1 pathways and glial responses	[178]
Epigenetic alterations	miR‐124	Reduced in epileptic tissue	Dysregulated in epilepsy cohorts; biomarker	Regulates neuronal identity genes and synaptic balance → excitability shift	[178]
Epigenetic alterations	HDAC1/2	Upregulated after SE; HDAC inhibition reduces epileptogenesis	Altered chromatin acetylation in epileptic tissue	Reduced histone acetylation represses neuroprotective and inhibitory gene expression	[74]

Abbreviations: AP, action potential; ASOs, antisense oligonucleotides; DEEs, developmental and epileptic encephalopathies; DRE, drug‐resistant epilepsy; FCD, focal cortical dysplasia; GAD, glutamic acid decarboxylase; GOF, gain‐of‐function; HDAC, histone deacetylases; HS, hippocampal sclerosis; LOF, loss‐of‐function; mTOR, mechanistic target of rapamycin; TLE, temporal lobe epilepsy.

Epileptogenesis, however, is rarely driven by a single pathway. Comparative studies indicate that genetic, inflammatory, metabolic, and structural mechanisms interact and evolve across disease stages. Neuroinflammation illustrates this convergence. In experimental models, activation of IL‐1β, HMGB1‐TLR4, and TNF‐α pathways lowers seizure threshold through modulation of synaptic transmission, receptor trafficking, and ion‐channel function [145]. Importantly, similar inflammatory signatures are detected in patients with DRE, including elevated cytokines, microglial activation, and BBB dysfunction in resected tissue and CSF (Table 1) [136, 145]. Metabolic and mitochondrial defects show a similar pattern. In animal models, ATP depletion, Na^+^/K^+^‐ATPase failure, and excessive ROS promote membrane depolarization, glutamate release, and network hypersynchrony [149]. Human studies provide parallel evidence, including interictal hypometabolism on FDG‐PET and enrichment of mTOR‐pathway or mitochondrial gene variants in focal cortical dysplasia and tuberous sclerosis‐associated epilepsy [149, 158, 189]. These findings support a model in which acute molecular injury is progressively converted into chronic network instability.

This evidence is beginning to change therapeutic thinking in epilepsy. It shows that treatment targets can extend beyond conventional suppression of neuronal firing. The clearest example is mTOR signaling. In models of FCD and tuberous sclerosis, mTOR inhibition reduces seizures and improves aspects of abnormal cortical development [145, 160, 190]. This mechanism has translated into clinical benefit, with everolimus producing meaningful seizure reduction in selected patients with tuberous sclerosis‐associated epilepsy [145, 160, 190]. Inflammatory pathways provide another therapeutic entry point. Experimental blockade of IL‐1 receptors signaling, HMGB1‐TLR4 signaling, or astrocytic TGF‐β pathways seizure development or reduces seizure severity in animal models, supporting ongoing efforts to modulate neuroinflammation in refractory epilepsy (Table 1). Epigenetic regulators are also emerging as candidate targets. miRNAs such as miR‐134 and chromatin‐modifying enzymes such as HDACs influence synaptic plasticity, neuronal survival, circuit maturation, and long‐term transcriptional remodeling (Table 1) [73, 191]. Together, these mechanisms suggest that future therapies should not only suppress seizures after they occur. They should also target upstream processes that initiate or stabilize epileptic networks. Combining preclinical causal evidence with clinical validation may help identify patients who are most likely to benefit from pathway‐targeted, immunomodulatory, metabolic, or epigenetic interventions. This approach moves epilepsy treatment toward mechanism‐guided therapy and, in selected contexts, toward disease modification.

## 4 Clinical Management

The clinical management of epilepsy aims to achieve sustained seizure control, minimize treatment‐related adverse effects, manage medical and neuropsychiatric comorbidities, and improve quality of life. ASMs remain the foundation of treatment. In many patients, they reduce seizure frequency and severity and provide effective symptomatic control. However, most ASMs act on downstream mechanisms of neuronal excitability, such as ion‐channel activity, synaptic transmission, or neurotransmitter release. They usually do not directly address the upstream disease processes that initiate or sustain epileptic networks. As a result, they do not reliably prevent epileptogenesis, halt long‐term disease progression, or reverse established network reorganization. Their effects on cognitive decline, psychiatric comorbidity, and broader disease burden are also limited [193]. These limitations underscore a major gap in current therapeutic in current epilepsy treatment. Conventional pharmacotherapy remains essential, but it should increasingly be complemented by strategies that target causal or disease‐sustaining mechanisms. Such approaches include etiology‐specific therapy, immunomodulation, metabolic intervention, pathway‐targeted treatment, neuromodulation, surgery, and emerging RNA‐ or gene‐based therapies. The long‐term goal is to move beyond symptomatic seizure suppression toward earlier intervention and, where possible, disease modification.

### 4.1 Diagnosis, Classification, and Work‐Up

#### 4.1.1 Diagnostic Evaluation and Seizure Classification

The evaluation of suspected epilepsy begins with a careful clinical history and detailed description of the event. Key elements include aura, seizure onset, motor and nonmotor manifestations, awareness, duration, injuries, autonomic signs, and postictal symptoms [194]. These features help define seizure type, localize the epileptogenic network, and guide etiological investigation. Eyewitness accounts and smartphone videos are often valuable, particularly for brief focal seizures, nocturnal events, and episodes that need to be distinguished from mimics [195]. Common differential diagnoses include syncope, transient ischemic attack, migraine aura, sleep disorders, movement disorders, and psychogenic nonepileptic seizures [196]. Potential provoking factors should also be documented, including sleep deprivation, fever, alcohol withdrawal, metabolic disturbance, infection, and recent medication changes, because they influence recurrence risk and the need for further evaluation.

After epileptic seizures are established, classification provides the basis for management and prognosis. The contemporary ILAE framework classifies seizures by onset as focal, generalized, or unknown. Focal seizures are further described according to awareness and motor or nonmotor onset [197, 198]. Epilepsies are then classified by epilepsy type and by etiology, including structural, genetic, infectious, metabolic, immune, and unknown causes. At the syndrome level, disorders such as JME, childhood AE, Lennox–Gastaut syndrome, and TLE are defined by characteristic combinations of age at onset, seizure type, EEG pattern, imaging features, comorbidities, and disease course [29]. Accurate classification has practical consequences. It guides ASM selection, informs prognosis, identifies candidates for surgery or neuromodulation, and determines when genetic, metabolic, autoimmune, or structural investigations are needed. Thus, diagnosis in epilepsy is not limited to confirming that seizures have occurred. It requires linking seizure semiology, electrophysiology, imaging, etiology, and syndrome diagnosis into a coherent clinical framework.

#### 4.1.2 Electrophysiological Assessment

Electrophysiological assessment is essential for the diagnosis and management of epilepsy. Routine scalp EEG is most commonly used to detect interictal epileptiform discharges, including spikes, sharp waves, spike–wave complexes, and polyspike–wave complexes. These findings are highly specific when correctly identified, but sensitivity is limited. A single routine EEG detects interictal epileptiform discharges in only 20%–50% of patients, and the yield is even lower after a first unprovoked seizure [194]. Therefore, a normal routine EEG does not exclude epilepsy. Accurate interpretation is critical. Benign variants, such as small sharp spikes, wicket spikes, and rhythmic temporal theta, may be mistaken for epileptiform discharges. Such errors contribute to epilepsy misdiagnosis, which is estimated to affect 20%–30% of cases [195]. When interpreted in the appropriate clinical context, routine EEG helps confirm the diagnosis, distinguish focal from generalized seizure types, and identify specific epilepsy syndromes such as AE and JME. These distinctions directly influence ASM selection.

When the diagnosis remains uncertain or events are clinically complex, prolonged video‐EEG monitoring is often required [199]. Long‐term video‐EEG allows simultaneous recording of behavior and brain activity. It is particularly useful for characterizing habitual events, refining seizure classification, distinguishing epileptic seizures from psychogenic nonepileptic seizures, and defining seizure onset patterns [200]. Ambulatory and home‐based EEG systems are also increasingly used. When combined with wearable sensors and machine‐learning algorithms, these tools permit longer real‐world monitoring, improve detection of unrecognized nocturnal seizures, and provide clinically useful information for treatment adjustment [201]. In drug‐resistant focal epilepsy, invasive electrophysiology may be needed for presurgical evaluation. Stereoelectroencephalography (SEEG) with depth electrodes or subdural grids provides high‐resolution mapping of seizure onset zones and propagation networks [202]. Integration with cortical stimulation and functional mapping enables individualized surgical planning, with the goal of maximizing seizure control while preserving eloquent cortex. Meta‐analyses indicate similar seizure‐freedom outcomes with SEEG and subdural grids, but lower infection risk and better access to deep and bilateral networks with SEEG, supporting its growing adoption in many epilepsy centers [203].

#### 4.1.3 Neuroimaging and Structural Evaluation

Neuroimaging is central to epilepsy evaluation, especially when a surgically remediable lesion is suspected. Contemporary 3 T epilepsy‐protocol MRI typically includes high‐resolution three‐dimensional T1‐weighted, T2‐weighted, and fluid‐attenuated inversion recovery sequences, together with coronal oblique acquisitions optimized for the hippocampus and regions of suspected pathology [204]. These protocols improve detection of hippocampal sclerosis, FCD, low‐grade tumors, vascular malformations, and developmental abnormalities such as polymicrogyria and heterotopia. High‐field and ultra‐high‐field MRI have further increased diagnostic yield. Standardized 7 T epilepsy protocols detect occult lesions that are occult on conventional imaging in patients with drug‐resistant focal epilepsy and may change clinical management in a substantial proportion of cases [205]. Parallel‐transmit 7 T systems also reduce susceptibility artifacts and improve visualization of subtle focal cortical dysplasia in some MRI‐negative patients. Beyond visual inspection, quantitative imaging techniques, including diffusion tensor imaging, diffusion kurtosis imaging, voxel‐based morphometry, and cortical thickness analysis, can reveal gray‐ and white‐matter abnormalities that are not apparent on routine MRI [206]. Automated morphometric postprocessing and machine‐learning‐based focal cortical dysplasia detection applied to 3 T epilepsy‐protocol MRI further improve lesion detection and presurgical localization in MRI‐negative focal epilepsy [207].

Functional neuroimaging complements structural approaches by delineating metabolic and hemodynamic features of epileptogenic networks. Interictal ^18^F‐FDG PET is widely used to localize focal hypometabolism in DRE, particularly TLE. Concordance among PET, MRI, and EEG is associated with higher rates of postoperative seizure freedom [189]. Ictal and interictal SPECT provides additional lateralization and localization in MRI‐negative or multilobar epilepsy [208]. Functional MRI is useful for both network characterization and presurgical mapping of language and memory, thereby reducing the need for invasive functional testing in selected patients [209]. Increasingly, clinical decision‐making relies on multimodal integration. Structural MRI, quantitative morphometry, PET or SPECT, magnetoencephalography or EEG source imaging, and SEEG provide complementary information about lesion location, seizure onset, propagation pathways, and eloquent cortex [210]. Experience from high‐volume epilepsy centers demonstrates that such integrated approaches improve lesion detection, enhance concordance with invasive recordings, and increase the precision of surgical planning, ultimately translating into improved seizure outcomes [211].

#### 4.1.4 Genetic Testing and Biomarkers

Genetic testing has become an important part of epilepsy evaluation, especially in patients with early‐onset seizures, severe developmental phenotypes, familial epilepsy, or no identifiable structural cause. Recent guidelines recommend genetic testing for DEEs, epilepsy with onset before 2 years of age, familial epilepsies, generalized epilepsies suggestive of channelopathies, and pharmacoresistant epilepsy without a clear lesion [212]. In DEEs, targeted gene panels and exome sequencing identify pathogenic or likely pathogenic variants in approximately 30%–50% of cases, often involving *SCN1A*, *SCN2A*, *KCNQ2*, *DEPDC5*, and *TSC1/2* [82]. Endorse exome or genome sequencing is therefore increasingly used as first‐tier test for unexplained epilepsy across age groups, reserving targeted panels for narrowly defined phenotypes [213].

A molecular diagnosis can directly change clinical management. Pediatric and adult studies demonstrate that genetic findings alter treatment or surveillance in 30%–60% of patients [214]. The clearest examples are genotype‐specific therapeutic decisions. In SCN1A‐related Dravet syndrome, sodium‐channel blockers may aggravate seizures and should be avoided. In mTORopathies due to *TSC1/2*, *DEPDC5*, or *MTOR* variants, mTOR inhibition with everolimus may reduce seizure burden in selected patients [192, 215, 216]. In GLUT1 deficiency, early initiation of ketogenic diet provides both antiseizure and disease‐modifying benefit [192, 215, 216]. Genetic diagnosis also informs prognosis, comorbidity surveillance, recurrence‐risk counseling, and reproductive decision‐making in families with highly penetrant variants. At the population level, PRS may further help stratify epilepsy susceptibility, although their routine clinical use remains limited [86].

Molecular biomarkers provide complementary information beyond genomic testing. Circulating and CSF markers of neuroinflammation and glial activation, such as GFAP, S100B, IL‐1β, and HMGB1, have been associated with seizure burden, cognitive impairment, and drug resistance in recent studies [217]. Markers of neuroaxonal injury, such as neurofilament light chain, may also reflect disease severity or ongoing neuronal damage. When combined with clinical phenotype, EEG, imaging, and genetic data, these biomarkers may support composite risk profiles. For example, inflammatory signatures could help identify patients more likely to benefit from immunomodulatory treatment [218]. Genomic testing and biomarker discovery are moving epilepsy evaluation beyond seizure classification alone. They help define etiology, identify actionable mechanisms, guide treatment selection, and improve long‐term risk stratification.

#### 4.1.5 Autoantibody Screening

Autoantibody testing and immune evaluation are important when autoimmune epilepsy is suspected. Evaluation should be considered in patients with subacute new‐onset seizures, new‐onset refractory status epilepticus, atypical neuropsychiatric features, cognitive decline, limbic hyperintensities on MRI, or poor response to ASMs [219]. In these settings, seizures may reflect immune‐mediated disruption of synaptic transmission, neuronal excitability, or limbic network function rather than primary structural epilepsy. Testing should include antibodies against neuronal surface antigens and selected intracellular antigens. Surface antibodies include LGI1, CASPR2, NMDAR, AMPAR, and GABABR antibodies [220]. Intracellular antibodies include GAD65, Hu, Ma2, and CV2 antibodies. These antibodies define distinct autoimmune encephalitis subtypes with different seizure phenotypes, tumor associations, treatment responses, and relapse risks. Surface antibodies are often directly pathogenic. NMDAR and AMPAR antibodies promote receptor internalization, whereas LGI1 and CASPR2 antibodies disrupt voltage‐gated potassium‐channel complex function and synaptic organization [221].

Accurate testing and cautious interpretation are essential. Both serum and CSF should be analyzed, because CSF testing improves diagnostic sensitivity and specificity, particularly for NMDAR and GABA_B_R antibodies and in selected LGI1 or CASPR2 cases [220]. Antibody positivity restricted to CSF is common in definite autoimmune encephalitis, whereas low‐titer serum antibodies alone may be nonspecific [222]. Current best practice favors cell‐based assays, often supported by tissue immunohistochemistry, to confirm antigen‐specific binding and reduce false‐positive results. Once autoimmune epilepsy is suspected or a pathogenic antibody is identified, early immunotherapy is critical. High‐dose corticosteroids, intravenous immunoglobulin, or plasma exchange are commonly used as first‐line therapies. Meta‐analyses and longitudinal cohorts indicate that early treatment is associated with favorable functional outcomes in approximately 70%–80% of patients and may reduce the risk of chronic epilepsy [223]. Second‐line therapies, including rituximab or cyclophosphamide, are recommended for refractory disease or high‐risk subtypes. Accumulating evidence supports rituximab in neuronal surface antibody‐mediated encephalitis and in relapse prevention for NMDAR and LGI1 encephalitis [224]. Long‐term management should include seizure monitoring, relapse surveillance, assessment of cognitive and psychiatric recovery, and tumor screening when paraneoplastic antibodies are present. Maintenance immunotherapy may be required in selected patients. Delayed or inadequate immunotherapy is associated with persistent seizures and progression to chronic epilepsy after autoimmune encephalitis [223]. Thus, timely recognition of autoimmune epilepsy can change the treatment strategy from symptomatic seizure suppression to mechanism‐based immune intervention.

Epilepsy diagnosis begins with structured clinical history and seizure classification according to current ILAE frameworks. Electrophysiological assessment then helps confirm epileptiform activity, define seizure type, and support syndrome diagnosis. Routine EEG, prolonged video‐EEG, ambulatory monitoring, and invasive recordings each provide different levels of information about seizure onset, propagation, and network involvement. Neuroimaging further refines anatomical and functional localization. Epilepsy‐protocol MRI, high‐field imaging, quantitative morphometry, PET, SPECT, functional MRI, and source‐localization techniques can identify structural lesions and delineate epileptogenic networks. When integrated with EEG and, when needed, SEEG, these tools guide surgical evaluation, ablation planning, and neuromodulatory targeting. Genetic testing, molecular biomarkers, and autoantibody screening add an etiological and mechanistic layer to diagnosis. They help stratify prognosis, identify treatable causes, and select patients for mechanism‐based therapies, including targeted metabolic, immune, genetic, or pathway‐directed interventions. Thus, modern epilepsy evaluation is moving from seizure description alone toward integrated diagnosis that links clinical phenotype, network localization, etiology, and individualized treatment.

### 4.2 Pharmacological Therapy

#### 4.2.1 Selection of First‐Line Antiseizure Medications

The choice of first‐line ASM is guided by seizure type, epilepsy syndrome, evidence of efficacy, tolerability, comorbidities, drug–drug interactions, and reproductive considerations. Updated ILAE evidence reviews support carbamazepine, phenytoin, lamotrigine, levetiracetam, and zonisamide as effective initial monotherapies for focal epilepsy, and valproate or ethosuximide for typical absence seizures [225]. Subsequent trials and guideline updates have refined these recommendations and placed greater emphasis on long‐term retention, adverse‐effect burden, cost‐effectiveness, and patient‐specific risk. For newly diagnosed focal epilepsy, lamotrigine and carbamazepine remain key first‐line options. Evidence from the SANAD trials and later meta‐analyses supports lamotrigine as a preferred initial therapy in many patients because of its favorable tolerability and cost‐effectiveness [226]. SANAD II Arm A further showed that lamotrigine had better treatment retention, seizure outcomes, and cost‐effectiveness than levetiracetam or zonisamide, arguing against routine first‐line use of the latter agents in newly diagnosed focal epilepsy [227]. Large real‐world cohorts, including studies from China, have reported similar findings, with higher long‐term retention and seizure remission rates for lamotrigine compared with carbamazepine, valproate, topiramate, or oxcarbazepine [228].

Other agents remain useful in selected clinical settings. Oxcarbazepine is an effective sodium‐channel blocker for focal epilepsy, but its use may be limited by hyponatremia and drug–drug interactions [229]. Levetiracetam may be preferred when rapid titration, minimal pharmacokinetic interaction, or reproductive safety is a priority. However, psychiatric adverse effects, including irritability, anxiety, and mood worsening, should be monitored [230]. Clobazam has been evaluated as monotherapy in limited studies, but current evidence is insufficient to support its routine use as a first‐line agent [228]. For generalized‐onset and unclassified epilepsies, valproate remains the most effective broad‐spectrum option in many patients. SANAD II Arm B showed that valproate was more effective and more cost‐effective than levetiracetam for achieving 12‐ and 24‐month seizure remission and for prolonging time to first seizure recurrence [231]. Accordingly, valproate remains a preferred first‐line therapy for IGEs in men and in women without childbearing potential [232]. When valproate is contraindicated, lamotrigine and levetiracetam are commonly recommended alternatives.

Reproductive safety is a major determinant of treatment selection. Because valproate carries a well‐established risk of teratogenicity and adverse neurodevelopmental outcomes, it should generally be avoided in women and girls of childbearing potential unless no suitable alternative is effective [233]. Recent WHO and international guidelines recommend lamotrigine or levetiracetam as first‐line options for women of childbearing age with focal or generalized‐onset seizures [234]. Emerging evidence suggests that levetiracetam may be more effective than lamotrigine in JME, whereas both agents appear broadly comparable in AE and generalized tonic–clonic seizures [234]. Thus, first‐line ASM selection increasingly requires matching syndrome‐level efficacy with individual safety, reproductive, psychiatric, and pharmacokinetic considerations.

#### 4.2.2 Mechanisms of Action Across Antiseizure Medication Classes

**FIGURE 6:**
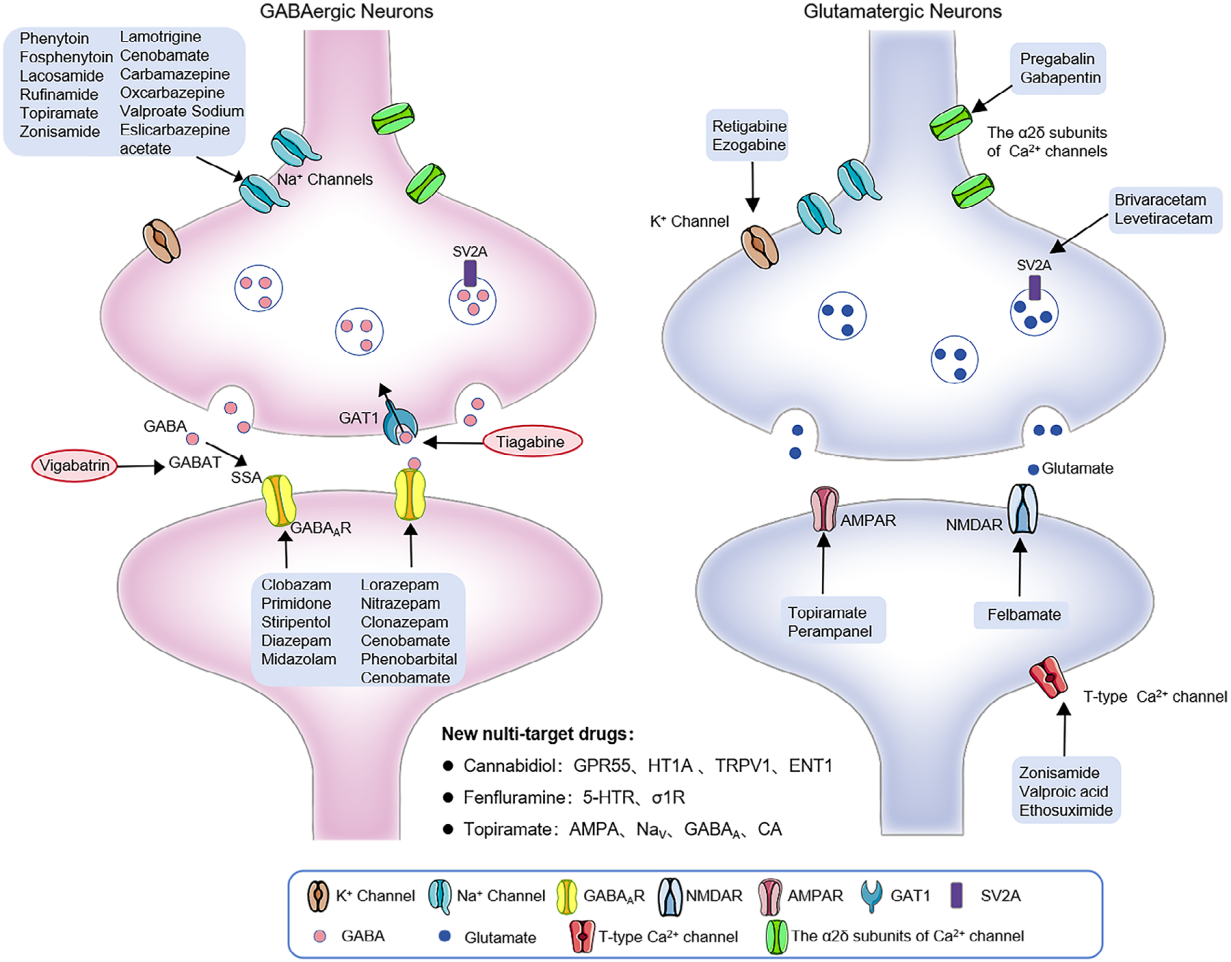
Molecular targets of clinically approved antiseizure medications. Antiseizure medications act through multiple mechanisms that converge on the control of neuronal excitability. These mechanisms can be grouped into four broad categories: modulation of voltage‐gated ion channels to alter intrinsic membrane excitability; enhancement of GABAergic inhibition by increasing GABA availability or potentiating GABA_A_ receptor function; suppression of AMPA or NMDA receptor‐mediated excitatory transmission; and modulation of synaptic vesicle release machinery.

Many ASMs act by reducing neuronal hyperexcitability through modulation of ion channels. Sodium‐channel blockers suppress pathological high‐frequency firing by promoting channel inactivation and limiting repetitive action‐potential generation (Figure 6) [235]. Classical agents such as carbamazepine, phenytoin, oxcarbazepine, eslicarbazepine, and lamotrigine primarily enhance fast inactivation, whereas lacosamide selectively promotes slow inactivation, thereby preferentially suppressing sustained depolarization and prolonged repetitive firing (Figure 6) [236]. Cenobamate further inhibits the persistent sodium current, which is a key contributor to paroxysmal depolarization shifts and burst firing (Figure 6) [235]. Calcium‐channel modulation provides another route to seizure control. Ethosuximide suppresses T‐type calcium channels in thalamocortical circuits and is particularly effective for typical absence seizures [237]. Gabapentin and pregabalin bind the α_2_δ subunit of high‐voltage‐activated calcium channels and reduce excitatory neurotransmitter release. Several broad‐spectrum ASMs also partially inhibit Cav2 channels, contributing to stabilization of synaptic transmission. Potassium‐channel activation further constrains neuronal excitability [235]. The Kv7 channel opener retigabine demonstrated that enhancement of the M‐current can hyperpolarize neuronal membranes and elevate firing thresholds, providing a foundation for future K^+^‐targeted therapies [238].

ASMs also modulate synaptic transmission (Figure 6). Levetiracetam and brivaracetam bind SV2A, and reduce vesicle release probability, with prominent effects at hyperactive synapses [239]. Enhancement of GABAergic inhibition remains a major therapeutic strategy. Benzodiazepines increase the frequency of GABA_A_ receptor channel opening, whereas barbiturates prolong channel open time, thereby strengthening inhibitory transmission [240]. Other agents act through related mechanisms. Stiripentol and cenobamate potentiate GABA_A_ receptor currents through distinct allosteric actions, whereas vigabatrin increases GABA availability by inhibiting GABA transaminase (Figure 6). Suppression of glutamatergic excitation is equally important. Perampanel selectively and noncompetitively antagonizes AMPA receptor, thereby reducing fast excitatory transmission. Felbamate inhibits NMDA receptor activity at the glycine‐binding site and also enhances GABAergic inhibition [241]. Additional mechanisms contribute to the broad pharmacology of several ASMs. Topiramate, zonisamide, and sulthiame, inhibit carbonic anhydrase isoenzymes, leading to pH‐dependent reductions in neuronal excitability (Figure 6) [242]. Fenfluramine exerts antiseizure and neuroprotective effects through modulation of serotonergic signaling and σ_1_‐receptor activity [243].

A further group of ASMs acts through disease‐specific or multimodal mechanisms. Cannabidiol has broad antiseizure effects by modulating intracellular calcium signaling, increasing extracellular adenosine via ENT1 inhibition, attenuating TRPV1 activity, and enhancing α_2_‐containing GABA_A_ receptor currents (Figure 6) [244]. Everolimus, an inhibitor of mTORC1, reduces seizure burden in tuberous sclerosis complex, and provides a clear example of pathway‐targeted therapy in a genetically defined epilepsy [235]. Several widely used ASMs display pronounced polypharmacology. Topiramate acts on sodium channels, GABA_A_ receptors, AMPA and kainate receptors, and carbonic anhydrase. Valproate enhances GABA synthesis, inhibits GABA degradation, modulates sodium and calcium channels, and influences epigenetic regulatory pathways (Figure 6) [245]. These broad actions may explain why such agents are effective across multiple seizure types, but also why they can produce diverse adverse effects. Together, these mechanisms show that seizure control rarely depends on a single molecular target. Effective therapy usually results from coordinated modulation of intrinsic membrane excitability, synaptic excitation and inhibition, transmitter release, metabolic signaling, and disease‐specific pathways. Mechanistic understanding of ASM action can therefore support rational drug selection, combination therapy, and the development of more targeted treatments for biologically defined epilepsy subgroups.

#### 4.2.3 Management of Drug‐Resistant Epilepsy

DRE is conventionally defined as failure of two appropriately chosen, adequately dosed, and well‐tolerated ASM regimens, whether used as monotherapy or in combination, to achieve sustained seizure freedom [246]. Approximately one‐third of people with epilepsy meet criteria for DRE, although estimates vary by population, healthcare setting, and epilepsy syndrome [247]. A 2021 meta‐analysis reported a pooled prevalence of 13.7% in community‐based cohorts and approximately 36% in clinic‐based samples, with cumulative incidence estimates of ∼25% in children and ∼15% in adults [248]. Despite the availability of more than 30 ASMs, the proportion of patients who develop DRE has changed little over recent decades. DRE is associated with .poorer prognosis, including increased risks of injury, SE, psychiatric and somatic comorbidities, premature mortality, particularly SUDEP [249].

Management begins by confirming true DRE. Pseudo‐resistance must be excluded because it may result from seizure misclassification, misdiagnosis, inappropriate ASM selection, subtherapeutic dosing, poor adherence, or persistent triggers such as sleep deprivation or alcohol use [250]. Once two adequate ASMs trials have failed, referral to a comprehensive epilepsy center is strongly recommended. This allows diagnostic reassessment, etiological clarification, and timely evaluation for surgery, neuromodulation, dietary therapy, immunotherapy, or other nonpharmacological interventions. Early referral is critical because delays in definitive treatment are associated with worse cognitive, psychosocial, and mortality outcomes [251].

When pharmacological treatment is continued, rational polytherapy should aim to improve seizure control while minimizing adverse effects. The guiding principle is to use the fewest necessary medications with complementary mechanisms of action. Combinations with overlapping mechanisms, such as multiple sodium‐channel blockers, should generally be avoided when adverse effects accumulate without clear benefit. Selected pairings, including valproate with lamotrigine or levetiracetam with a sodium‐channel blocker, have been associated with higher responder rates or better tolerability in some studies [249]. Polytherapy should be introduced stepwise, with predefined treatment goals, attention to pharmacokinetic interactions, and regular reassessment of benefit. Regimens should be simplified when additional agents fail to improve seizure control or cause unacceptable toxicity. Optimal management of DRE therefore requires more than adding successive medications. It requires mechanism‐informed pharmacotherapy, early identification of treatable etiologies, timely consideration of surgical or neuromodulatory options, and long‐term management of adherence, comorbidities, safety, and quality of life [252].

### 4.3 Epilepsy Surgery

Epilepsy surgery is an effective treatment for selected patients with drug‐resistant focal epilepsy. Presurgical evaluation aims to define the epileptogenic zone as precisely as possible while estimating the risk of postoperative neurological or cognitive deficits. Standard evaluation includes high‐resolution epilepsy‐protocol MRI, interictal EEG and prolonged video‐EEG monitoring, and detailed neuropsychological testing [253]. Many centers also use FDG‐PET, ictal or interictal SPECT, magnetoencephalography, and task‐based or resting‐state functional MRI to improve localization and identify eloquent cortex [254]. When noninvasive findings are discordant, or when the suspected epileptogenic focus lies near eloquent cortex, SEEG provides millimeter‐scale mapping of seizure onset and propagation to guide resective, disconnective, or ablative treatment [255].

Resective surgery remains the most effective intervention for focal DRE when a well‐defined epileptogenic zone can be safely removed. Anterior temporal lobectomy, selective lesionectomy, and tailored extratemporal resections achieve long‐term seizure freedom in approximately 60%–80% of patients with mesial TLE, hippocampal sclerosis, FCD type II, cavernous malformations, or low‐grade tumors when complete resection is feasible [256]. Successful surgery is often associated with improved quality of life, greater functional independence, reduced medication burden, and better long‐term survival. Disconnective procedures are used when seizure networks cannot be treated by focal resection alone. Corpus callosotomy is mainly considered for patients with disabling drop attacks, whereas hemispherotomy is used in selected children with catastrophic hemispheric epilepsies or epileptic spasms [257]. Contemporary series show elimination of drop attacks in about 50%–55% of patients after callosotomy [258, 259]. Hemispherotomy can achieve seizure freedom or near‐freedom in more than 60%–70% of appropriately selected children and may stabilize or improve developmental outcomes [258, 259].

Minimally invasive surgical techniques have expanded treatment options for patients who are poor candidates for open resection. MRI‐guided laser interstitial thermal therapy (LITT) is increasingly applied in mesial TLE, hypothalamic hamartoma, periventricular nodular heterotopia, and cavernous malformations [260]. Prospective and multicenter studies report Engel Class I outcomes in approximately 60%–70% of mesial TLE cases and seizure remission rates exceeding 50% across other lesional substrates [261]. Compared with open surgery, LITT is associated with reduced perioperative morbidity, shorter hospital stays, and better preservation of cognitive function, particularly verbal memory in dominant temporal procedures [262]. confirm the durable value of epilepsy surgery. A meta‐analysis encompassing more than 16,000 surgical cases demonstrated that approximately two‐thirds of patients achieved sustained seizure freedom or near‐freedom for at least 1 year, substantially outperforming continued medical therapy [263]. Longitudinal cohorts further report seizure‐freedom rates of 50%–60% at 5–10 years following temporal lobe resections, with somewhat lower but clinically meaningful outcomes after extratemporal surgery [261]. MRI‐negative epilepsy, multifocal epileptogenicity, and incomplete resection predict poorer outcome. Nonetheless, successful surgery is consistently linked to lower SUDEP risk, reduced overall mortality, and better cognitive, psychosocial, and vocational improvement [264]. These findings argue against viewing epilepsy surgery as a last resort. For appropriately selected patients with drug‐resistant focal epilepsy, surgical evaluation should be considered early as part of mechanism‐guided and network‐informed care.

### 4.4 Neuromodulation

Neuromodulation is an established treatment option for patients with DRE who are not suitable candidates for curative resection or ablation. This includes patients with nonlocalizable seizures, multifocal epilepsy, bilateral epileptogenic networks, or seizure onset within eloquent cortex. Three invasive neuromodulatory modalities are approved for adult DRE, vagus nerve stimulation (VNS), deep brain stimulation of the anterior nucleus of the thalamus (ANT‐DBS), and responsive neurostimulation (RNS) [265]. Treatment selection is increasingly based on seizure type, network anatomy, comorbidities, prior treatment response, and surgical risk rather than a fixed stepwise algorithm. These interventions are primarily palliative. Sustained seizure freedom is uncommon, but many patients achieve clinically meaningful improvement. Across studies, more than half of treated patients reach a responder outcome, usually defined as a reduction in seizure frequency of at least 50%, often with improvement in quality of life [266]. Neuromodulation is therefore most often considered after comprehensive presurgical evaluation has shown that curative resection or ablation is unlikely, unsafe, or incomplete, and when further medication escalation is unlikely to provide sufficient benefit.

VNS is the most widely utilized neuromodulatory therapy, and has more than two decades of clinical experience. Its effects are mediated through afferent vagal projections to the nucleus tractus solitarius, monoaminergic brainstem nuclei, thalamocortical circuits, and limbic networks [267]. These pathways may reduce epileptiform synchronization and modulate network excitability. VNS is approved for both adults and children aged 4 years and older, including patients with Lennox–Gastaut syndrome [268]. Randomized trials demonstrated short‐term seizure reductions of approximately 25%–30% with high‐output stimulation compared with 6%–15% with low‐output settings [269]. Long‐term meta‐analyses including more than 3000 patients report responder rates of 40%–60%, with sustained improvements in mood, alertness, and overall quality of life [270]. ANT‐DBS targets a key thalamic relay in focal and secondarily generalized epilepsies and uses continuous stimulation to disrupt pathological network synchronization. In the SANTÉ trial, long‐term follow‐up showed a median seizure reduction of about 75% at 10 years, with acceptable safety and possible reduction in SUDEP risk [271]. RNS provides closed‐loop, activity‐triggered stimulation delivered directly to one or two seizure foci. Unlike continuous stimulation, it detects epileptiform activity and delivers stimulation in response to patient‐specific electrographic patterns. Nine‐year prospective data reveal progressive and durable seizure reduction, reaching a median reduction of approximately 75%, with stable or improved cognitive outcomes [272]. RNS is especially useful for bilateral mesial TLE or seizure foci located in eloquent cortex, where resection carries unacceptable risk.

Noninvasive neuromodulation is being explored as an adjunctive strategy for DRE, rather than as a replacement for established invasive therapies. Transcutaneous VNS, repetitive transcranial magnetic stimulation (rTMS), and transcranial direct current stimulation (tDCS) have shown seizure‐reducing or network‐modulating effects in small trials, although none is currently approved for epilepsy [273]. A recent meta‐analysis reported a seizure frequency reduction of approximately 40%–45% over 4–8 weeks, accompanied by suppression of IEDs and few serious adverse events [274]. Evidence supporting rTMS and transcutaneous VNS remains less definitive. Interpretation is limited by small sample sizes, heterogeneous stimulation protocols, variable epilepsy syndromes, and limited long‐term follow‐up [267]. Future development will require standardized protocols, biomarker‐guided patient selection, and durable outcome data. Emerging priorities include minimally invasive or noninvasive closed‐loop systems, wearable and machine‐learning‐enabled adaptive stimulation, and individualized targeting of seizure networks. Alternative targets, including the centromedian thalamus and hippocampus, are also being investigated for generalized, multifocal, or cognitively complex epilepsies. These advances aim to improve patient stratification, enhance therapeutic efficacy, and extend neuromodulation toward more precise long‐term seizure management.

### 4.5 Dietary and Lifestyle Interventions

Dietary and lifestyle interventions are important adjunctive in the comprehensive management of epilepsy. These approaches do not replace pharmacological, surgical, or neuromodulatory therapies, but can complement them by targeting metabolic, inflammatory, and network‐level processes that influence seizure susceptibility [275]. Among dietary interventions, the KD, modified Atkins diet (MAD), and low‐glycemic index treatment (LGIT) is the most extensively studied. Each differs in dietary restrictiveness, tolerability, and suitability for specific patient groups [276]. Lifestyle modifications, including regular sleep, physical activity, stress reduction, and avoidance of individual seizure triggers, can further support seizure control and improve quality of life.

Ketogenic and related dietary therapies are established options for DRE. Randomized and quasi‐randomized trials demonstrate that children treated with KDs are approximately three times more likely to achieve seizure freedom and up to six times more likely to achieve at least a 50% reduction in seizure frequency compared with usual care [277]. Adults also derive benefit, although adherence and discontinuation rates are higher. Meta‐analyses indicate that several forms of ketogenic dietary therapy are effective in the short term. The MAD appears to have comparable efficacy with better tolerability than the classical KD in some patients, making it a practical dietary option for many clinical setting [278]. Recent pooled analyses confirm that MAD combined with ASM increases the likelihood of achieving at least a 50% seizure reduction by more than sixfold [279]. Large contemporary cohorts report that 50%–60% of patients experience at least a 50% reduction in seizure frequency and 10%–20% achieve seizure freedom [280]. Responses are particularly strong in GLUT1 deficiency and selected mTORopathies, where dietary therapy is mechanistically aligned with impaired brain energy metabolism or growth‐signaling abnormalities.

Mechanistic studies have begun to clarify how dietary therapies reduce seizures. Their effects are associated with increased ketone bodies, altered cerebral energy metabolism, modulation of excitatory and inhibitory neurotransmission, and changes in gut microbiome‐derived metabolites [281]. A 2024 prospective study further identified microbial taxa and serum metabolomic signatures associated with treatment response, suggesting that future dietary therapy may be guided by biological markers rather than applied uniformly to all patients [282]. Adherence remains a major limitation, especially in adults. Gastrointestinal symptoms, dyslipidemia, restricted food choice, social inconvenience, and long‐term dietary fatigue commonly lead to discontinuation. Less restrictive approaches, including the MAD, LGIT, and intermittent or time‐restricted ketogenic protocols, may improve tolerability while preserving therapeutic benefit in selected patients [283]. Current evidence supports early consideration of dietary therapy in DRE, particularly when the underlying biology suggests metabolic vulnerability. Careful monitoring of growth, bone health, lipid metabolism, renal complications, and nutritional adequacy is essential.

Lifestyle interventions also influence seizure susceptibility and long‐term outcome. Structured aerobic exercise and combined endurance‐resistance programs improve physical fitness, mood, cognition, and quality of life without increasing seizure frequency in most patients [284]. A meta‐analysis of 14 exercise studies showed consistent benefits across psychosocial and functional domains, with a trend toward seizure reduction [285]. Recent trials further support the safety of aerobic and resistance training, and regular moderate‐to‐vigorous physical activity may reduce long‐term mortality risk in people with epilepsy [286]. Sleep assessment should be integrated into routine epilepsy care. A 2024 population‐based study reported clinically significant sleep problems in nearly half of adults with epilepsy, and these problems were strongly associated with poorer seizure control and reduced quality of life [287]. Thus, comprehensive epilepsy management should include dietary therapy when appropriate, structured physical activity, systematic sleep evaluation, and individualized lifestyle counseling alongside optimized medical, surgical, and neuromodulatory treatment.

### 4.6 Precision and Emerging Disease‐Modifying Therapies

Precision medicine in epilepsy aims to move treatment from empirical seizure suppression toward mechanism‐based intervention. Advances in genetics, neuroimmunology, systems neuroscience, and biomedical engineering now make it possible to classify some epilepsies according to genotype, pathway biology, immune status, and disease course. This approach can guide etiology‐specific treatment, including targeted pharmacotherapy, dietary therapy, immunotherapy, neuromodulation, and gene‐based strategies. Monogenic epilepsies, mTORopathies, metabolic epilepsies, and immune‐mediated epilepsies are particularly suited to this framework [288]. However, disease modification requires more than identifying a target. It also depends on early etiological diagnosis, reliable biomarkers, and natural‐history data that define when neural circuits remain responsive to intervention.

RNA‐ and gene‐based therapies extend this concept by directly targeting transcript‐level or gene‐level defects in monogenic epilepsies. ASOs can modify splicing, reduce toxic transcripts, or increase expression of haploinsufficient genes. In Dravet syndrome, SCN1A‐targeted ASOs, such as STK‐001, are designed to increase expression from the functional allele and restore Na_v_1.1‐dependent interneuron excitability [289, 290]. Similar approaches are being developed to reactivate the paternal UBE3A allele in Angelman syndrome [288]. Other RNA‐based strategies are also emerging. RNA interference may selectively silence gain‐of‐function transcripts in genes such as KCNT1, SCN2A, or DEPDC5, whereas RNA‐editing platforms offer the possibility of reversible correction of pathogenic transcripts without permanent genomic alteration [291]. In parallel, AAV‐based gene therapies have achieved durable seizure reduction in preclinical focal epilepsy models by increasing expression of engineered potassium channels or neuropeptide Y receptor systems [292]. These approaches provide a high degree of mechanistic specificity and may be especially relevant for severe genetic epilepsies that remain refractory to conventional treatment.

Mechanism‐based immunotherapy is an important component of precision treatment in autoimmune epilepsy. Early use of corticosteroids, intravenous immunoglobulin, or plasma exchange can reduce pathogenic antibody burden and suppress acute neuroinflammation [293]. Timely treatment may reverse antibody‐mediated synaptic dysfunction before irreversible network injury develops. Across cohorts, early immunotherapy is associated with better seizure control, improved cognitive outcomes, and a lower risk of chronic epilepsy. Second‐line therapies provide additional immune control in refractory disease. Rituximab has shown benefit in LGI1‐ and CASPR2‐mediated disease, whereas cyclophosphamide, tocilizumab, and bortezomib are generally reserved for refractory autoimmune encephalitis or new‐onset refractory status epilepticus [224]. Treatment selection is increasingly guided by antibody subtype, disease stage, relapse risk, and the presumed immune mechanism.

Pathway‐targeted pharmacological therapy provides another route to mechanism‐based care. In mTOR‐driven epilepsies such as tuberous sclerosis complex, everolimus produces sustained seizure reduction and can reduce tumor burden, supporting the clinical value of targeting the causal pathway rather than only suppressing seizures [288]. Experimental studies further suggest that mTOR inhibition may attenuate abnormal network growth and inflammatory signaling [9]. Other pathway‐based strategies are emerging. Targeting mutant IDH1 pathways may reduce glioma‐associated seizures and alter the local immune environment. In chronic epilepsy models, inhibition of JAK‐STAT3 signaling reduces spontaneous seizures and neuroinflammatory markers [294, 295]. These examples suggest that selected epilepsies may be treated by targeting the biological processes that sustain seizure networks.

Biomarker‐guided therapy aims to match treatment to measurable disease mechanisms. Autoantibody profiles already help guide immunotherapy choice and relapse monitoring [296]. Circulating microRNAs, serum metabolomic signatures, and inflammatory markers are being evaluated for identifying epileptogenic tissue, estimating recurrence risk, and stratifying patients for targeted interventions [297]. Electrophysiological biomarkers, including high‐frequency oscillations and network coherence, can support surgical planning and neuromodulation programming [298]. Continuous intracranial recordings from RNS devices provide patient‐specific biomarkers that may guide adaptive stimulation and medication adjustment [299]. Structural and functional neuroimaging further help identify candidates for surgery or neuromodulation and may enable longitudinal monitoring of disease progression [300].

Together, these advances are shifting epilepsy care toward mechanism‐informed treatment. Antibody‐guided immunotherapy, pathway‐targeted pharmacology, RNA‐ and gene‐based therapies, and biomarker‐informed strategies all share the same principle: treatment should be matched to the biological driver of disease whenever possible. This approach does not eliminate the need for symptomatic seizure control, but it expands the therapeutic goal toward earlier intervention, better stratification, and, in selected contexts, disease modification.

## 5 Future Directions and Conclusions

This review presents epilepsy as a common, heterogeneous, and chronically evolving brain network disorder. Its burden is shaped by marked epidemiological disparities, diverse etiologies, extensive comorbidity, and persistently increased risks of disability and premature death. Seizure expression is also temporally organized. Circadian, multiday, and seasonal rhythms influence seizure probability across syndromes, indicating that temporal biology is relevant to both disease mechanisms and clinical management. At the mechanistic level, genetic susceptibility, ion‐channel and synaptic dysfunction, neuroinflammation, metabolic and mitochondrial stress, epigenetic remodeling, and structural‐connectomic reorganization interact across disease stages. These processes destabilize E/I balance, promote epileptogenesis, and help sustain drug resistance. These advances have, in turn, begun to reshape clinical care, informing precision diagnostics, biomarker development, surgery and neuromodulation strategies, immunotherapy, dietary and lifestyle interventions, and emerging pathway‐, RNA‐, and gene‐directed treatments. The central task is now to connect epidemiology, mechanism, and treatment into a practical model that enables earlier intervention, biologically stratified care, and disease modification.

Despite these advances, major challenges remain. Approximately one‐third of people with epilepsy continue to have drug‐resistant seizures despite the availability of multiple ASMs. Comorbidity is also pervasive. Recent clinic‐based cohorts show that nearly 80% of adults with epilepsy have at least one chronic comorbidity, and persistent seizures nearly double this burden, contributing to poorer quality of life and premature mortality [40]. These findings emphasize that seizure control alone is not sufficient. Long‐term management must also address psychiatric, cognitive, sleep‐related, cardiovascular, metabolic, and other systemic comorbidities. Global inequity further amplifies the burden of epilepsy. Nearly 80% of people with epilepsy live in LMIC, where treatment gaps often exceed 50%–75% because of limited access to specialists, essential medicines, diagnostic infrastructure, and social support, as well as persistent stigma [301]. Thus, progress in epilepsy care cannot rely on pharmacological innovation alone. Meaningful improvement will require integrated strategies that address biological heterogeneity, comorbid disease, early referral, diagnostic access, treatment affordability, and structural inequities at the same time.

Within this evolving landscape, precision medicine represents an important but still incompletely realized direction for epilepsy care. Its central aim is to match treatment to the biological mechanism, clinical context, and disease stage of each patient. This requires integration of genomic testing, molecular biomarkers, imaging, electrophysiology, and deep phenotyping, rather than reliance on seizure classification alone. At present, the strongest clinical examples are limited to genetically or mechanistically defined epilepsies. In tuberous sclerosis complex, everolimus is approved for TSC‐associated focal seizures and provides proof of principle that pathway‐directed therapy can improve seizure control in selected patients [216]. Ketogenic therapy is similarly established for *GLUT1* deficiency syndrome, where treatment is directly aligned with impaired brain glucose transport and energy metabolism [280]. Beyond these validated examples, many mechanism‐based approaches remain at earlier stages of translation. In neuro‐oncology, mutant IDH inhibition has entered late‐stage clinical practice for *IDH*‐mutant glioma and has shown exploratory benefits on seizure control. However, its role remains context‐specific and indirect from the perspective of epilepsy therapeutics [294]. By contrast, targeting inflammatory pathways such as JAK‐STAT or HMGB1 remains largely preclinical or early translational [295]. Biomarker development is also advancing. Multiomics integration can link genotype to phenotype and identify candidate markers for prognosis, recurrence risk, and treatment stratification [15]. Yet most signatures remain exploratory and require validation in prospective, multicenter cohorts before they can guide routine clinical care.

A parallel shift is occurring in therapeutic development. The field is moving gradually from incremental symptomatic suppression toward interventions that more directly address causal mechanisms. Small molecules still dominate the epilepsy pipeline, but several mature programs now extend beyond conventional sodium‐channel blockers and GABAergic modulators [302]. The Kv7 channel opener XEN1101 has advanced to Phase 3 clinical trials, whereas preferential modulation of persistent sodium current with PRAX‐562 is being evaluated in Phase 2 studies for *SCN2A*‐ and *SCN8A*‐related DEEs [302]. However, clinical translation remains uncertain. Soticlestat reached Phase 3 testing in Dravet syndrome and Lennox–Gastaut syndrome but failed to meet its primary endpoints, leading to discontinuation of development in 2025 [303]. This outcome illustrates both the promise and the high attrition rate of mechanism‐based drug development. Gene‐based therapies are also progressing, although their maturity varies widely [26]. AAV‐delivered transcriptional activation of *SCN1A* with ETX101 has entered Phase 1/2 studies in Dravet syndrome, showing that gene‐regulatory strategies are now clinically testable in humans [302]. In contrast, allele‐specific editing, base editing, and prime editing remain largely preclinical despite encouraging results in cellular and animal models [158]. Thus, the most balanced view is that epilepsy therapeutics are moving toward earlier, biomarker‐informed, and potentially disease‐modifying intervention. However, only a small subset of these approaches has so far reached established clinical use or late‐stage development. Continued progress will depend on early diagnosis, robust biomarkers, natural‐history studies, scalable trial designs, and careful assessment of long‐term safety and developmental outcomes.

Concurrently, data‐driven technologies are also reshaping epilepsy monitoring and long‐term care, although their clinical maturity differs across applications. AI‐enabled seizure detection is more advanced than seizure forecasting. EEG‐based systems and wearable multimodal platforms have shown growing value for real‐time or near‐real‐time event detection [304]. By contrast, seizure forecasting remains less mature. Models based on long‐term scalp EEG, intracranial recordings, and wearable biosignals have shown encouraging performance in retrospective datasets and selected prospective studies, but external validation, calibration across diverse populations, and workflow integration remain major barriers [305]. In the near term, AI is likely to be most useful as clinical decision support rather than as fully autonomous seizure prediction. Promising applications include automated EEG analysis, multimodal phenotyping, presurgical evaluation, risk stratification, and longitudinal tracking of patient‐level trajectories [304]. Over time, integration of forecasting algorithms with RNS, adaptive stimulation paradigms, and digital biomarkers may support a shift from reactive seizure treatment toward anticipatory management [265]. However, clinical implementation will require prospective validation, interpretability, regulatory clarity, and evidence that these tools improve patient outcomes rather than only model performance.

Temporal biology provides another opportunity to improve epilepsy care. Seizures often show circadian, multiday, and seasonal patterns, suggesting that treatment may be optimized by aligning therapy with periods of increased seizure vulnerability. Chronotherapy applies chronopharmacological principles to match ASM dosing with rhythms in drug exposure, target sensitivity, and seizure risk. Clinical observations support this concept in selected settings. Evening‐weighted dosing of phenytoin, clobazam, or carbamazepine has been associated with reduced nocturnal seizure burden, and bedtime diazepam can reduce spike–wave activity in specific syndromes [306, 307, 308]. Preclinical studies provide mechanistic support but also highlight translational limitations. In acute and chronic pilocarpine models, tiagabine administered at ZT6 produces greater seizure suppression and longer latency to seizure onset and generalized seizures than dosing at ZT18 [309]. Valproate and phenytoin also show time‐dependent differences in anticonvulsant efficacy and neurotoxicity in rodent models, partly due to circadian variation in plasma exposure and central nervous system sensitivity [310]. However, rodents are nocturnal, and their clock phase, sleep architecture, and drug metabolism differ from those of humans [311]. Direct extrapolation is therefore difficult. Human evidence for chronotherapy remains promising but incomplete. Benefits of time‐adjusted dosing have been modest or inconsistent in some studies, suggesting that chronotherapy may be most useful in patients with clear circadian seizure clustering, sleep‐sensitive epilepsies, or predictable nocturnal seizures [312]. Wider implementation is limited by the lack of robust randomized trials stratified by epilepsy type, seizure timing, sleep phenotype, and circadian phase [306, 307]. Individual differences in circadian timing, age, lifestyle, comorbid sleep disorders, polypharmacy, and adherence further complicate standardized dosing schedules. Future studies should combine long‐term seizure diaries, wearable or intracranial recordings, and pharmacokinetic‐pharmacodynamic modeling to personalize dosing schedules. Chronotherapy is therefore a biologically plausible strategy, but its clinical value will depend on rigorous validation and careful patient selection.

In conclusion, epilepsy is a heterogeneous but tractable brain network disorder shaped by interacting genetic, synaptic, metabolic, immune, and epigenetic mechanisms. Clinical care is now moving beyond broad seizure suppression toward mechanism‐informed diagnosis, biomarker‐guided stratification, pathway‐targeted therapy, neuromodulation, digital monitoring, and gene‐ or RNA‐based intervention. The central challenge for the coming decade is to translate these advances into earlier, safer, and more equitable care, with the long‐term goal of preventing epileptogenesis, reducing disability, and achieving disease modification in biologically defined patient groups.

## Author Contributions

**Tianpeng Zhang and Jian Liu** conceived and designed the project. **Tianpeng Zhang, Jian Liu, and Xinyan Wu** drafted the manuscript. **Jian Liu, Xinyan Wu, Yu Yang, Zinan Liu, and Jiabin Yu** analyzed the data and drafted the manuscript. **Tianpeng Zhang, Jian Liu, Xinyan Wu, Ting Yin, Feining Huang, and Manqi Kong** participated in the revision of the manuscript. All the authors have read and approved the final manuscript.

## Funding

This work was supported by the Guangdong Province Basic and Applied Basic Research Project (No. 2023B1515020047), Scientific Research Platforms and Projects of Guangdong Higher Education Institutions (No. 2025ZDZX2011), and University‐Hospital Joint Fund Project of Guangzhou University of Chinese Medicine (GZYBA2024XKG04).

## Ethics Statement

The authors have nothing to report.

## Conflicts of Interest

The authors declare no conflicts of interest.

## Data Availability

All data generated and/or analyzed during the current study are included in this published article.
